# Adsorption kinetics of methylene blue from wastewater using pH-sensitive starch-based hydrogels

**DOI:** 10.1038/s41598-023-39241-z

**Published:** 2023-07-24

**Authors:** Fatemeh Mohammadzadeh, Marzieh Golshan, Vahid Haddadi-Asl, Mehdi Salami-Kalajahi

**Affiliations:** 1 Department of Polymer Engineering and Color Technology, Amirkabir University of Technology, Tehran, Iran; 2 Faculty of Polymer Engineering, Sahand University of Technology, P.O. Box 51335-1996, Tabriz, Iran; 3 Institute of Polymeric Materials, Sahand University of Technology, P.O. Box 51335-1996, Tabriz, Iran

**Keywords:** Environmental chemistry, Chemistry, Materials science

## Abstract

In this work, starch/poly(acylic acid) hydrogels were synthesized through a free radical polymerization technique. The molar ratios of acrylic acid to *N*,*N*′-methylenebisacrylamide were 95:5, 94:6, and 93:7. The samples exhibited an amorphous porous structure, indicating that the size of the pores was contingent upon the amount of cross-linking agent. The quantity of acrylic acid in structure rose with a little increase in the amount of the cross-linking agent, which improved the hydrogels’ heat stability. The swelling characteristics of the hydrogels were influenced by both the pH level and the amount of cross-linking agent. The hydrogel with a ratio of 94:6 exhibited the highest degree of swelling (201.90%) at a pH of 7.4. The dominance of the Fickian effect in regulating water absorption in the synthesized hydrogels was demonstrated, and the kinetics of swelling exhibited agreement with Schott's pseudo-second order model. The absorption of methylene blue by the hydrogels that were developed was found to be influenced by various factors, including the concentration of the dye, the quantity of the cross-linking agent, the pH level, and the duration of exposure. The hydrogel 95:5 exhibited the highest adsorption effectiveness (66.7%) for the dye solution with a concentration of 20 mg/L at pH 10.0. The examination of the kinetics and isotherms of adsorption has provided evidence that the process of physisorption takes place on heterogeneous adsorbent surfaces and can be explained by an exothermic nature.

## Introduction

Researchers have become interested in starch-based hydrogels because of wide range of useful properties including biocompatibility, biodegradability, physiochemical properties, high efficiency, and reasonable price^1^. Hydrogels derived from starch are utilized in a wide range of industries including the environment, agriculture, medicine, absorbents, and so forth^2^. Dye adsorption by these hydrogels in industrial wastewater is one of their many potential uses that has garnered a lot of attentions. Organic dyes are used in numerous industries, including textiles, prints, plastics, and cosmetics, but they are extremely hazardous to water supplies and ecosystems^3^. Aromatic organic dyes, like methylene blue (MB), are more toxic and stable than other types of organic dyes; they can also cause nausea, vomiting, tissue necrosis, and nerve damage in humans^4^. Dye separation can be accomplished through a variety of processes, including sedimentation^5^, ion exchange^6^, coagulation^7^, filtration^8^, and adsorption^9, 10^. Adsorbents are superior to other methods by having advantages such as recyclability, low cost, high absorption efficiency, flexibility in performance, efficiency and simple handling^11^.

Despite the different advantages of starch-based hydrogels, weaknesses such as low mechanical resistance and high sensitivity to degradation have prevented their widespread use in industry^12^. Starch can be modified via physical^13^, chemical^14^, blending^15^, and enzymatic^16^ techniques to solve the aforementioned issues. Oxidation^17^, hydrolysis^18^, esterification^19^, and grafting^20^ are among the methods of modifying starch chemically. Chemical grafting with vinyl monomers such as acrylic acid has gained interest due to the precise control over the modification process, greater adsorption capacity, and creating a more stable and uniform product^21^. Furthermore, grafting hydrogel with poly(acrylic acid) (PAA) improves properties for instance mechanical strength, thermal resistance, and ability to absorb water, as well as broadening its application^22, 23^. Introducing negative charges to starch is essential for the adsorption of ions or positively-charged dyes by starch-based hydrogels. Starch oxidation and modification with poly(acrylic acid) are two methods used for this purpose^24^. While starch oxidation may alter the starch structure and gelation properties, modification of starch with poly(acrylic acid) offers advantages such as a simplified process, enhanced water absorption capacity, tunable properties, and improved stability. This method effectively reduces costs and addresses potential environmental concerns associated with the modification process^25, 26^. Several factors, such as temperature, pH, dye concentration, and crosslinking agent can influence the adsorption efficiency of hydrogels^27^. The addition of *N,N*′-methylenebisacrylamide (MBA) as a crosslinker to starch hydrogel brings numerous benefits, including the facilitation of a stable structure formation, improved structural integrity, enhanced water absorption capacity, and the ability to tailor porosity, swelling behavior, and mechanical properties^28^. This versatility makes the hydrogel an exceptional choice for diverse applications in agriculture, biomedicine, and beyond^29^.

Introducing an innovative and sustainable method, this study synthesizes starch-based hydrogels grafted with poly(acrylic acid), offering a cost-effective and eco-friendly approach. This sustainable approach provides simplicity and affordability, making it highly practical for large-scale production. The investigation delves into the fascinating characteristics of these hydrogels, exploring their unique swelling behavior, adsorption kinetics, and isotherms. Remarkably, the results unveil their remarkable potential as exceptionally efficient adsorbents for dye removal. By optimizing key factors, including cross-linking agent concentration, these hydrogels demonstrate enhanced performance, opening doors for various applications in wastewater treatment and environmental remediation. This research sheds light on the intricate mechanisms of dye adsorption, advancing the field of sustainable materials. Merging principles from green chemistry, materials science, and environmental engineering, the study propels the utilization of starch-based hydrogels as eco-friendly alternatives with exceptional adsorption capabilities, revolutionizing the treatment of hazardous organic dyes in industrial wastewater.

## Experimental section

### Materials

Starch from potato, ceric ammonium sulfate (CAS, ≥ 94%), *N,N*′-methylenebisacrylamide (MBA, 99%), acrylic acid (AA, 99%), and methylene blue (MB) were purchased from Sigma-Aldrich.

### Preparation of starch/PAA hydrogels

A single-necked flask was filled with starch (1 g), distilled water (5 mL), and MBA and stirred under nitrogen at ambient temperature. The reaction solution was mixed with CAS as initiator, and AA, and the temperature was raised to 70 °C. Finally, the mixture was poured into a teflon mold and reaction was continued for 48 h at 60 °C. To evaluate the influence of the amount of crosslinker on the final properties, three samples, S-AA-1, S-AA-2, and S-AA-3 were prepared with different molar ratios of AA to MBA (95:5, 94:6, and 93:7, respectively). The weight ratio of starch and acrylic acid was 1:1. The selection of the 1:1 starch to acrylic acid ratio in our hydrogel synthesis was driven by the objective of balancing the structural integrity and sustainability of the gel. Alterations in the amount of acrylic acid had a direct impact on the concentration of the cross-linking agent, MBA, as it depends on the acrylic acid content. Decreasing the amount of acrylic acid resulted in reduced MBA concentration, leading to insufficient firmness and a propensity for collapse in the resulting hydrogel. Conversely, increasing the amount of acrylic acid posed a challenge to the sustainability of the hydrogel. To strike a compromise between gel stability and sustainable properties, we opted for the 1:1 ratio as the most suitable choice.

### Characterizations

Fourier-transform infrared spectroscopy (FTIR) of hydrogels combined with potassium bromide pellets was investigated in the 400–4000 cm^−1^ range using a Bruker Tensor 27 FT-IR Spectrometer manufactured in Germany. Using an X-ray diffractometer (EQUINOX3000, Inel, France) with 40 kV voltage, 30 mA current, and Cu Kα radiation, the crystallinity characteristic was examined. Under a nitrogen atmosphere, samples were heated from 25 to 600 °C at a heating rate of 10 °C/min to ascertain their thermal stability using thermogravimetric analysis (TGA) (Q600, TA, USA). Field Emission Scanning Electron Microscope (FE-SEM) (MIRA3 FEG-SEM, Tescan, Czech) operating at 30 kV was utilized to examine the morphology of the synthesized samples. The water-swollen samples were freeze-dried for 48 h using a freeze dryer (Martin Christ, Germany). The samples were then covered in gold to make them more conductive. The size of hydrogel pores was determined using ImageJ software. A portion of the synthesized samples was placed in 100 mL distilled water with specific pH values to examine swelling properties. Four pH values were specifically chosen to investigate the swelling and absorption behavior of starch/poly(acrylic acid) hydrogels: an acidic pH of 2.0, a pH close to the pK_a_ of acrylic acid (4.8), a neutral pH of 7.4, and an alkaline pH of 10.0. At specific intervals, samples were weighed and measurement was repeated until the sample weight was fixed. The swelling ratio percentage was calculated with Eq. (1)^30^.

1 $$Q=\frac{{M}_{t}-{M}_{0}}{{M}_{0}}\times 100$$

where *Q*, *M*_*t*_ and *M*_*0*_ are the percentage of water absorption (g_water_/g_absorbent_), sample weight at time t (g), and initial sample weight (g), respectively.

### Study of MB adsorption utilizing prepared starch-based hydrogels

To evaluate the effectiveness of hydrogel in absorbing MB at room temperature and pH values of 2.0, 4.8, 7.4, and 10.0, 0.05 g of hydrogel was immersed in MB solution. At certain intervals, 1.5 mL of the aqueous solution was removed and replaced with distilled water. MB solution was made at concentrations of 2, 3, 4, 5, 10, and 20 mg/L to investigate the effect of dye concentration on adsorption kinetics. A UV/visible spectrophotometer (Hanon, China) at 665 nm for pH 2.0, 663 nm for pH 4.8, 665 nm for pH 7.4, and 667 nm for pH 10.0 was utilized for determining MB concentration. Hydrogel absorption capacity (q_e_, mg dye/g hydrogel) and dye adsorption efficiency (E, %) were calculated using Eqs. (2) and (3), respectively^21^.

2 $${\mathrm{q}}_{\mathrm{e}}=\frac{({\mathrm{C}}_{\mathrm{i}}-{\mathrm{C}}_{\mathrm{e}})\times \mathrm{V}}{\mathrm{m}}$$

3 $$\mathrm{E}=\frac{({\mathrm{C}}_{\mathrm{i}}-{\mathrm{C}}_{\mathrm{e}})}{{\mathrm{C}}_{\mathrm{i}}} \times 100$$

where C_i_, C_e_, V, and m are the initial concentration of MB solution (mg/L), the concentration of the MB solution after adsorption (mg/L), the volume of the solution (mL), and the mass of the adsorbent (g), respectively.

To further comprehend the adsorption mechanism, the adsorption kinetics were investigated using pseudo-first order (PFO), pseudo-second order (PSO), and intraparticle diffusion (IPD) models, as indicated by Eqs. (4)^31^, (5)^32^, and (6)^33^.

4 $$Ln \left({q}_{e}-{q}_{t}\right)={Lnq}_{e}-{k}_{1}\times t$$

5 $$\frac{t}{{q_{t} }} = \frac{1}{{q_{e}^{2} \times k_{2} }} + \frac{t}{{q_{e} }}$$

6 $$q_{t} = k_{3} \times t^{0.5} + C$$

where *q*_*e*_, *q*_*t*_, *t*, *k*_*1*_, *k*_*2*_, *k*_*3*_, and *C* are respectively the adsorption capacity of the adsorbent in the equilibrium state (mg/g), the adsorption capacity of the adsorbent at time t (mg/g), time (h), the rate constant of the PFO equation (h^−1^), the rate constant of the PSO equation (g/(mg h)), the rate constant of the IPD ((mg/g)/h), and a constant associated with the thickness of the boundary layer (mg/g). Furthermore, the initial adsorption rate (mg/(g h)) in the PSO model can be determined using Eq. (7)^34^.

7 $$h= {k}_{2}\times {q}_{e}^{2}$$

Langmuir (Eq. (8))^35^, Freundlich (Eq. (9))^36^, and Temkin (Eq. (10))^37^ adsorption isotherms were analyzed using experimental data to define the interaction between the adsorbed substance and the adsorbent.

8 $$\frac{{C}_{e}}{{q}_{e}}=\frac{1}{{K}_{L}\times {q}_{m}}+\frac{{C}_{e}}{{q}_{m}}$$

9 $$Ln{q}_{e}=\frac{1}{n}\times Ln{C}_{e}+Ln{K}_{F}$$

10 $${q}_{e}=\left(\frac{R\times T}{b}\right)\times Ln{K}_{T}+(\frac{R\times T}{b})Ln{C}_{e}$$

where *C*_*e*_*, q*_*e*_, *q*_*m*_, *K*_*L*_, *n*, *K*_*F*_, *R*, *T*, *b*, and *K*_*T*_ are the concentration of MB solution in the equilibrium state (mg/L), the adsorption capacity of the adsorbent in the equilibrium state (mg/g), the maximum capacity of adsorption (mg/g), the Langmuir adsorption constant (L/mg), the Freundlich dimensionless constant indicating adsorption intensity, the Freundlich adsorption constant (mg/g), the temperature (K), the heat of adsorption (kJ/mol) and the Temkin adsorption constant (L/g), respectively.

## Result and discussion

**Figure 1:**
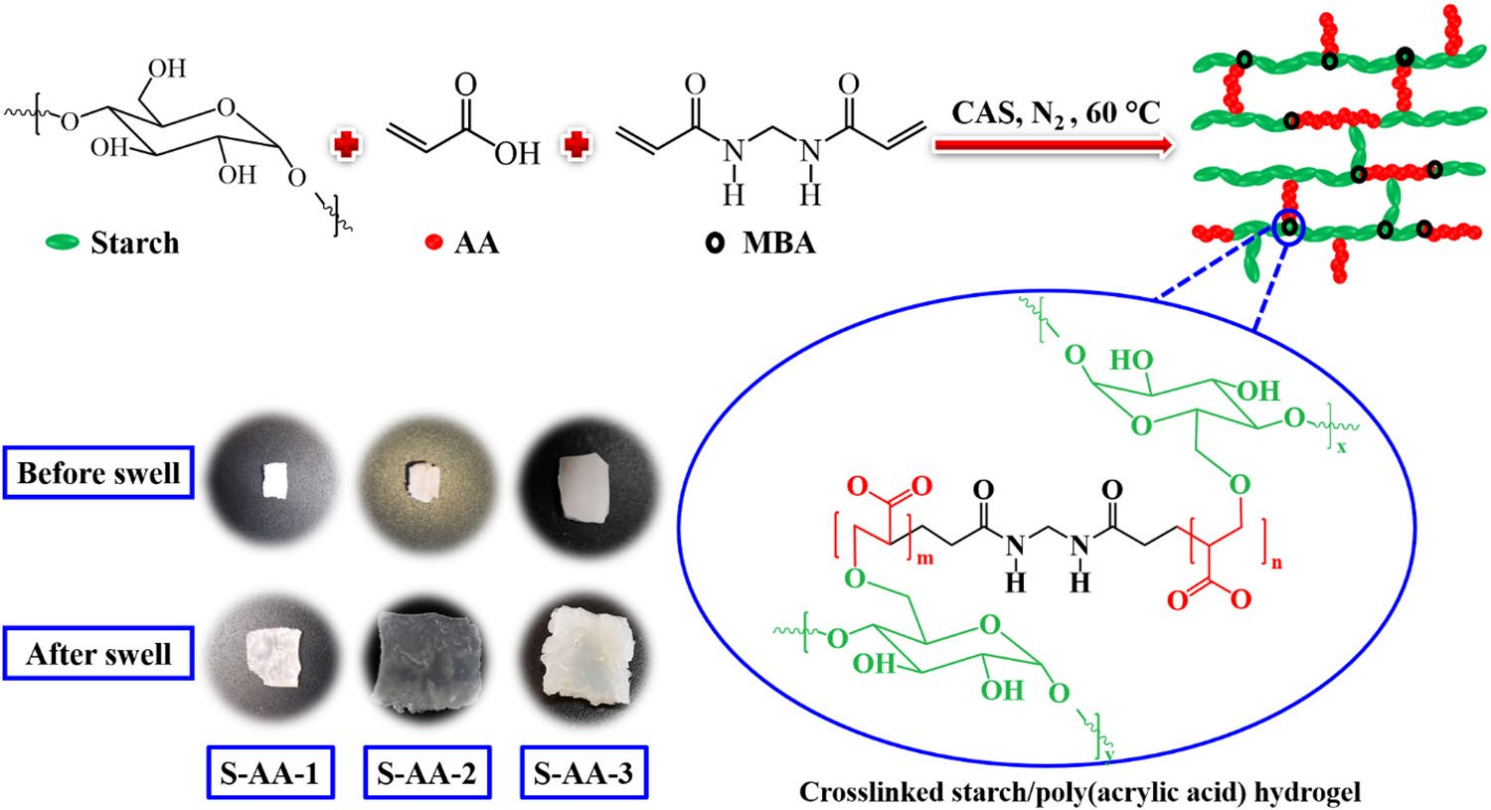
Synthesis of starch/PAA-based hydrogels.

Herein, starch/PAA-based pH-sensitive adsorbents with different amounts of crosslinker have been prepared by solution graft polymerization of PAA onto starch using CAS as initiator and MBA as crosslinker. Figure 1 depicts the polymerization mechanism to prepare hydrogels.

### Characterization of starch-based hydrogels

**Figure 2:**
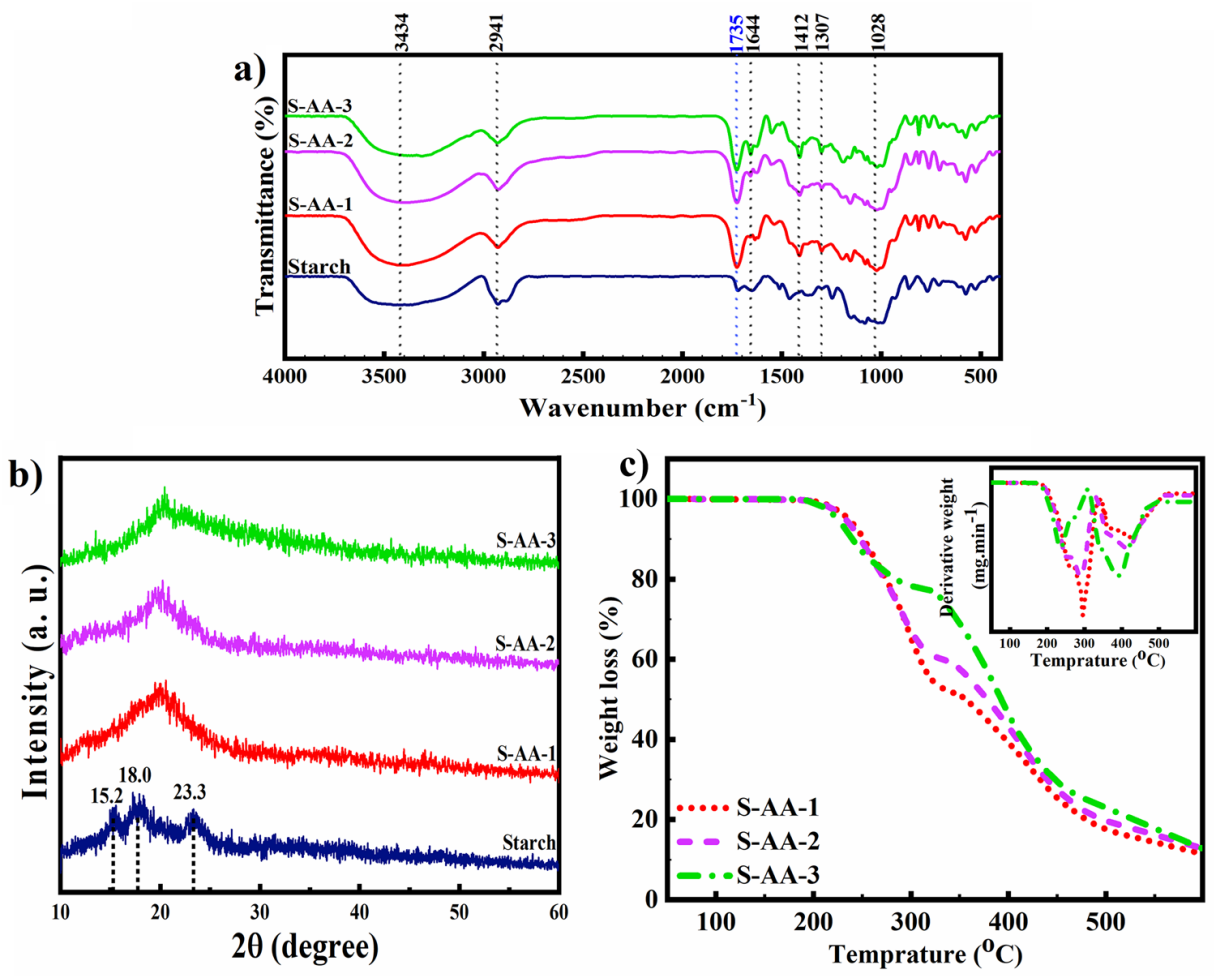
FTIR spectra*,* XRD patterns, and TGA thermograms of starch and starch/PAA hydrogels.

Evaluation and confirmation of hydrogels based on starch were performed using FT-IR, XRD, TGA, and FE-SEM analyses. Figure 2a depicts the FTIR spectra of starch and starch/PAA-based hydrogels. The individual peaks of starch occurred at wavenumbers of 3434 cm^−1^, 1028 cm^−1^, and 1644 cm^−1^, corresponding to O–H stretching, C–O–C asymmetric stretching vibration, and C–O bending associated with OH group, respectively. Furthermore, the peaks appearing at 1307 cm^−1^, and 1412 cm^−1^ are related to C–H angular^38^. In the hydrogel samples, in addition to the peaks of starch, a peak at 1735 cm^−1^ is attributed to the stretching of the carbonyl groups of PAA which implies that PAA has successfully bonded to starch. At 2941 cm^−1^, C–H stretching of PAA and starch was also detected^39^. Figure 2b depicts the XRD patterns of starch and synthesized hydrogels. Starch characterization peaks were at 2θ = 15.2° (101), 18.0° (100), and 23.3° (110). To assess the effect of the degree of crosslinking during the synthesis of different hydrogel samples, a comparison of the percentage of crystallinity between starch and PAA-based hydrogels was performed using XRD. The percentage of crystallinity of pure starch was determined to be 37.8% using the ratio between the area under the diffraction peaks and the total area of the entire XRD pattern. In the XRD pattern of starch/PAA-based hydrogels, a broad peak without starch characterization peaks was observed, indicating the amorphous structure of the hydrogel samples^40^. The researchers also reported the disruption of the starch crystal structure through the process of PAA grafting onto starch^41^. Because of intramolecular and intermolecular hydrogen bonding, starch has a highly ordered crystalline structure. The steric hindrance of similar charges generated by the interaction of starch OH groups with COO^−^ of PAA limited the mobility of starch chains in hydrogel samples. As a result, the formation of intermolecular hydrogen bonds in starch was reduced, causing the starch crystal structure to disintegrate and the crystalline phase to be destroyed^42^.

TGA was utilized to assess thermal stability (Fig. 2c, Table 1). The analysis indicates a two-stage degradation of hydrogels. The first step of decomposition at 182–320 °C is related to breaking the C–O–C bonds of the main chain of starch and the dehydration of the saccharide ring. The second degradation stage is associated with the decomposition of PAA grafted to starch at 320–520 °C^43^. The percentage of grafted acrylic acid was determined using Eq. (11) and reported in Table 1^44^:

11 $$\mathrm{Acrylic \; acid \; ratio}(\mathrm{\%})= \frac{{\mathrm{W}}_{1}}{{\mathrm{W}}_{1}+{\mathrm{W}}_{2}}\times 100$$

where W_1_ and W_2_ are weight loss in stage 1 and stage 2, respectively. By increasing the amount of crosslinking agent, the amount of grafted acrylic acid to the hydrogel structure was increased. The drop in WL_max_ and increase in T_d50_ with increasing crosslinking agent indicated a partial enhancement of in thermal stability which was caused by the increased activation energy required to destroy samples with higher crosslinking agents^45^.

**Table 1:** TGA results of starch/PAA-based hydrogels.

Sample	T_d10_ (°C)^a^	T_d50_ (°C)^b^	WL_max_ (%)^c^	PAA ratio (%)
S-AA-1	250.3	345.2	88.5	43.4
S-AA-2	246.4	377.6	87.2	50.5
S-AA-3	239.4	392.1	86.9	69.4

^a^Temperature referring to 10% weight loss.

^b^Temperature referring to 50% weight loss (°C).

^c^Maximum weight loss.

**Figure 3:**
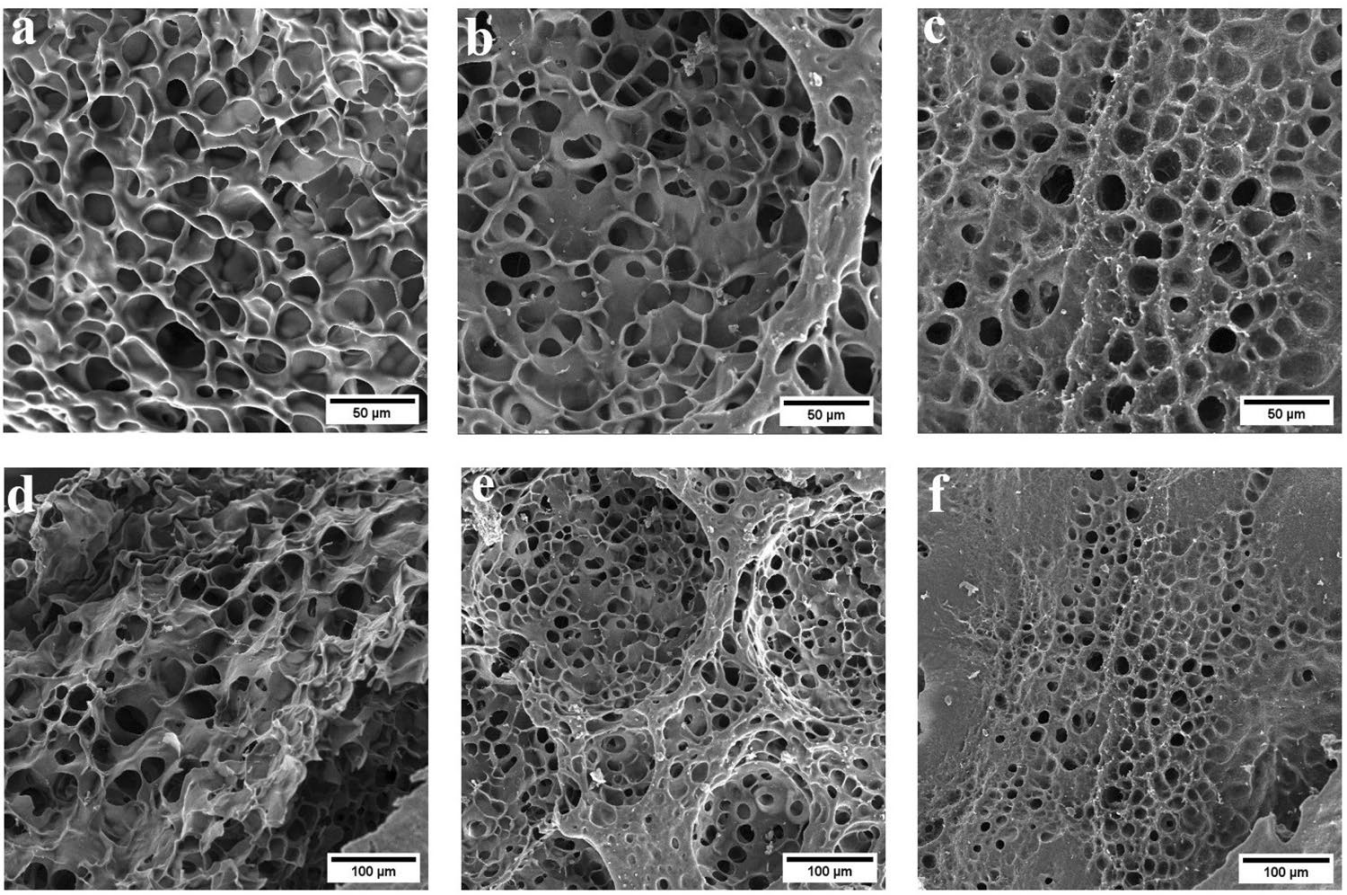
FE-SEM images of the surface of hydrogel samples S-A A-1 (**a**,**d**), S-AA-2 (**b**,**e**), and S-AA-3 (**c**,**f**) at ×500 and ×250 magnification scale.

The bulk morphology of the hydrogels were examined using FE-SEM (Fig. 3). All three samples demonstrated the porous structure which provides sites for solution diffusion into the polymer network and interaction of external stimuli with the polymer network. Grafting of PAA to starch enhances surface roughness and creates folds. Folds have a higher specific surface area than starch, facilitating water diffusion into the polymer network^46^. The average size of pores in the S-AA-1, S-AA-2, and S-AA-3 samples was 22.9, 17.1, and 14.5 µm, respectively, indicating that pore size decreased with increasing crosslinker which is attributed to increased hydrogel elasticity, which depends on the crosslinking density^47^. Moreover, existence of larger pores decreases transportation resistance and enhances water absorption capacity^48^.

### Swelling of hydrogels

**Figure 4:**
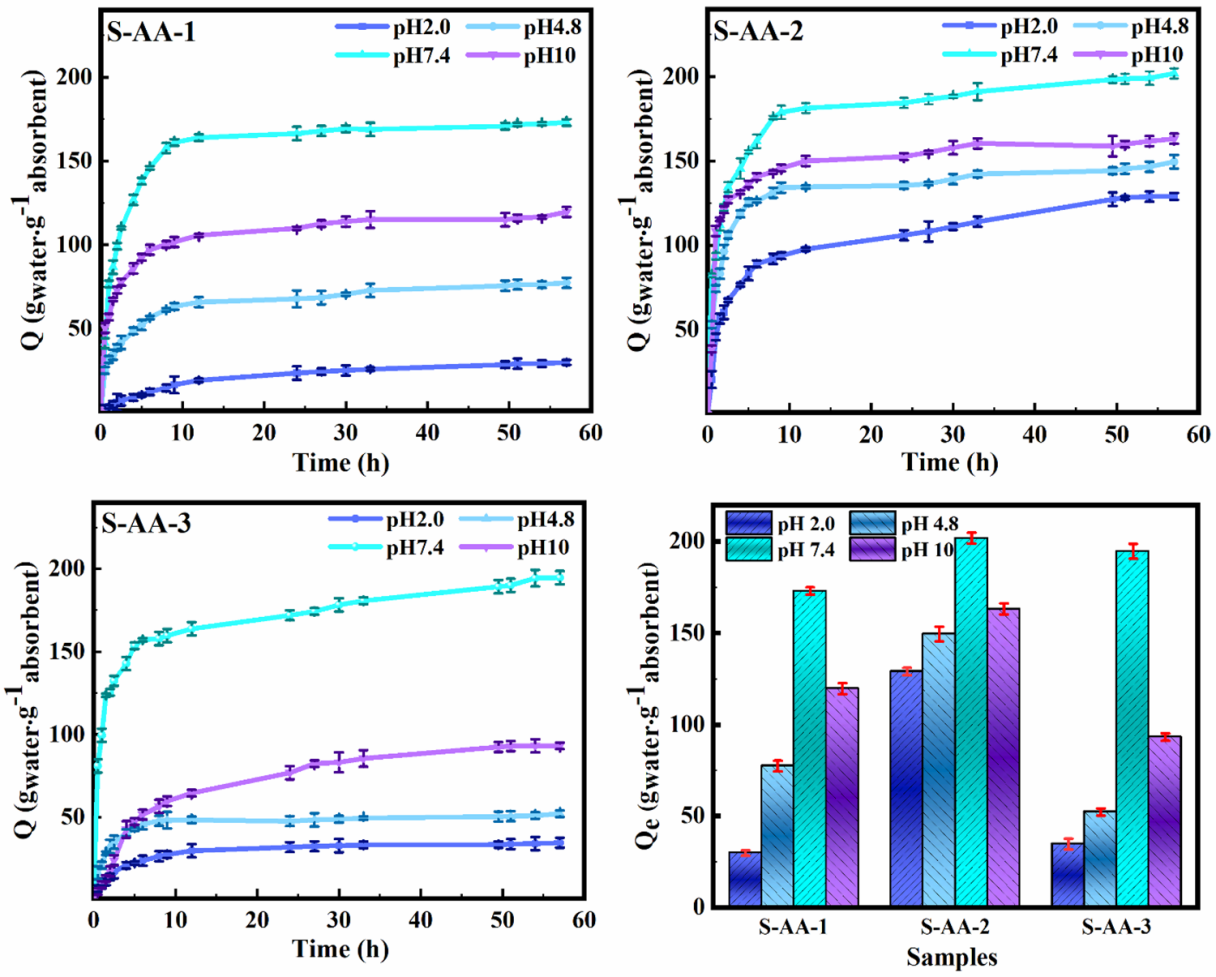
Swelling properties of starch/PAA hydrogels at pH 2.0, 4.8, 7.4, and 10.0.

Figure 4 illustrates the equilibrium swelling (Q_e_) and swelling behavior of the samples across a range of pH values. All the samples showed a dramatic swelling in the initial times and then reached equilibrium. The pH affects the behavior of ionic hydrogels. In the synthesized samples, grafting of PAA to starch caused in the induction of a significant number of negatively-charged COO^−^ groups. The results revealed that swelling increased as the pH varied from 2.0 to 7.4 whereas it decreased at pH = 10.0 and maximum swelling was observed at pH = 7.4. Carboxylate groups are protonated in an acidic environment, resulting in hydrogen bonding between polymer chains which act like physical crosslinks and lead to polymer network shrinkage and decreased swelling. Deprotonation of the carboxylic groups occurs when the pH is greater than PAA's p*K*_*a*_ which results in swelling due to electrostatic repulsions between chain segments^49^. At pH = 10.0, protecting Na^+^ cations in NaOH solution from COO^−^ groups inhibited the complete anion-anion repulsion in hydrogels, resulting in reduced water absorption^50^. The observed trend regarding the impact of pH on the swelling behavior aligns with existing literature. However, it should be noted that the hydrogels synthesized in this study exhibited a notably higher rate of swelling^51, 52^. Furthermore, a comparison of Q_e_ of S-AA-1 and S-AA-2 at every pH revealed that Q_e_ increased at higher crosslinking agent concentration. Increased cross-linking results in the formation of a three-dimensional structure in the polymer network, allowing water to permeate the hydrogel structure more effectively^53^. However, comparing Q_e_ for S-AA-2 with S-AA-3 showed that Q_e_ decreased with increasing crosslinker due to increased crosslinking density among polymer chains, which causes the creation of a stiffer structure that prevents more water from entering the structure^53^.

The modified power law equation, Eq. (12), was used to understand the water transport mechanism into the hydrogel^52^.

12 $$\frac{{M}_{t}}{{M}_{e}}=k\times {t}^{n}$$

where *M*_*t*_ and *M*_*e*_ are hydrogel weight at time t (g) and maximum hydrogel weight (g), respectively. The *k* is a proportionality constant, and *n* is a description of the type of diffusion mechanism. The equation is limited to 60% of absorbed water, which corresponds to a linear response of water absorption to swelling time. Type of water diffusion in the hydrogel is identified based on the range of *n*. *n* is calculated and given in Table 2 by plotting ln (*M*_*t*_*/Me*) versus time. Results indicated that for all the synthesized hydrogels *n* is < 0.5, indicating Fickian diffusion, which signifies that diffusion is the water absorption control mechanism. Schott's pseudo-second order swelling kinetic model, (Eq. (13)), was used to determine the swelling kinetics from results obtained^54^.

13 $$\frac{t}{{S}_{t}}=\frac{1}{{k}_{is}}+\frac{t}{{S}_{th}}$$

where *S*_*t*_, *S*_*th*_, and *k*_*is*_ are the swelling ratio at time* t*, the maximum theoretical swelling ratio, and the initial swelling ratio constant, respectively. By plotting *t/S*_*t*_ versus *t* and calculating the slope and intercept, *Q*_*th*_ and *k*_*is*_ were calculated (Table 2). The pseudo-second order swelling kinetic model governs the swelling procedure, as evidenced by the excellent agreement of theoretical *Q*_*th*_ data with experimental data and the achievement of R^2^ > 0.9800. The variation of *k*_*is*_ with pH is similar to the variation of *Q* with pH and is determined by the rate of relaxation of polymer chains in the polymer network. The ionization of carboxylate groups and electrostatic repulsions occurred as the pH increased from 2.0 to 4.8, leading to enhanced polymer chain relaxation. This phenomenon increased with increasing pH up to 7.4. Rapid relaxation facilitates the ability for water molecules to permeate the polymer network, thereby accelerating the rate of swelling. At pH = 10.0, the decrease in the mobility of polymer chains due to the screening effect of Na^+^ cations resulted in a decline in water diffusion into the polymer network; as a result, *k*_*is*_ decreased^54^.

**Table 2:** Swelling kinetics and diffusion parameters of starch/PAA hydrogels at various pH levels.

Sample	pH	Diffusion parameters		Kinetics parameters			
		n	R^2^	Q_e_	Q_th_	k_is_	R^2^
S-AA-1	2.0	0.05	0.9515	29.8	33.9	0.001	0.9861
	4.8	0.09	0.9856	77.3	78.4	0.006	0.9979
	7.4	0.20	0.9959	173.0	176.0	0.024	0.9997
	10.0	0.11	0.9900	119.6	119.3	0.016	0.9993
S-AA-2	2.0	0.15	0.9637	129.0	132.1	0.007	0.9933
	4.8	0.15	0.9878	149.5	148.5	0.022	0.9995
	7.4	0.13	0.9617	201.9	194.7	0.030	0.9990
	10.0	0.08	0.9752	163.2	163.5	0.022	0.9978
S-AA-3	2.0	0.06	0.9852	34.7	35.7	0.002	0.9983
	4.8	0.09	0.9973	52.2	51.9	0.008	0.9992
	7.4	0.15	0.9470	194.7	202.8	0.025	0.9991
	10.0	0.16	0.9786	93.2	105.6	0.002	0.9989

### MB adsorption kinetics through hydrogels

**Figure 5:**
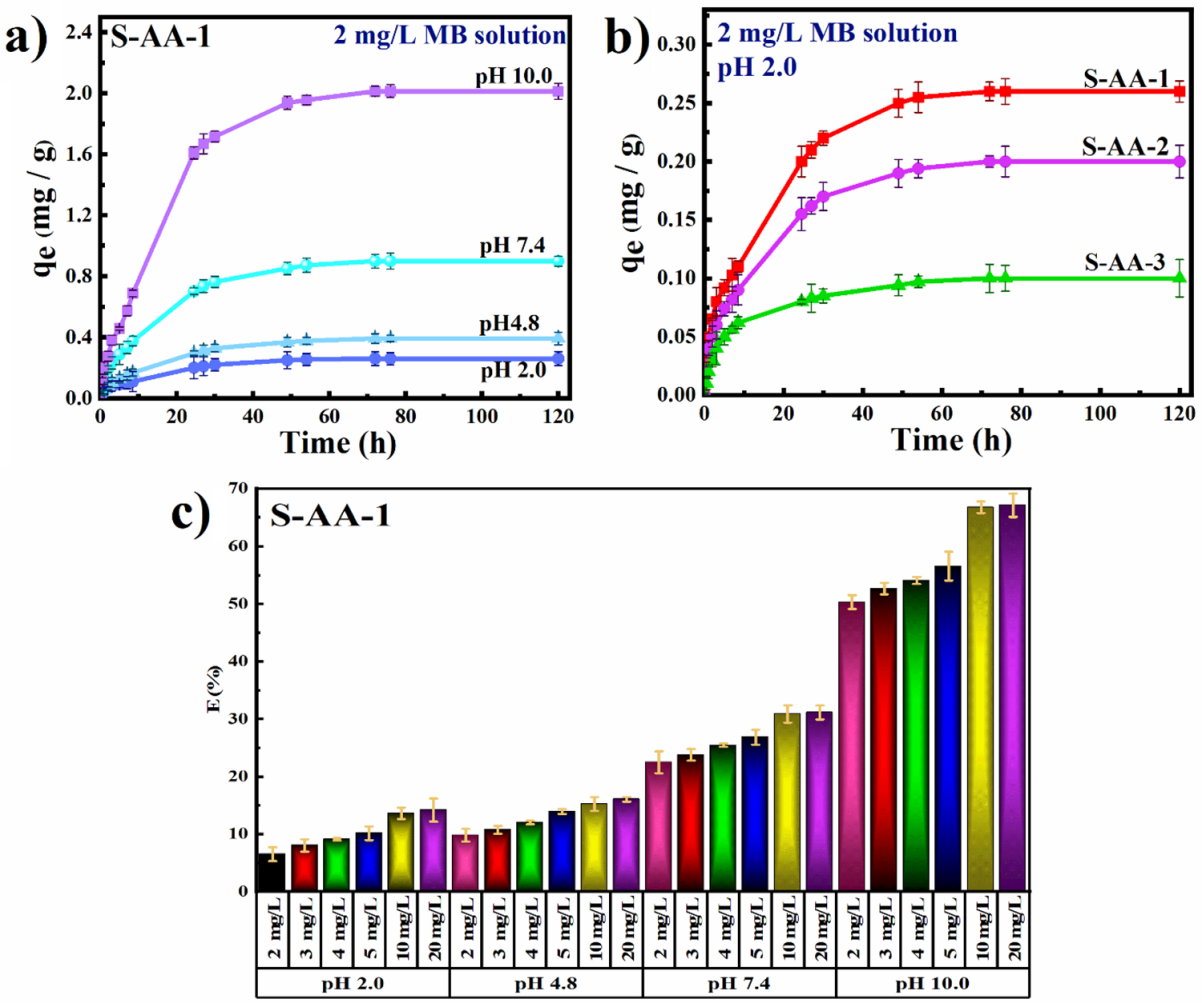
(**a**) The influence of pH on S-AA-1 adsorption capacity, (**b**) The influence of crosslinking agent on the adsorption capacity of synthesized hydrogels, (**c**) The influence of the initial dye concentration on the adsorption efficiency of hydrogel S-AA-1.

MB is widely used as a textile dye, but its hazardous contamination poses a concern to human health and safety. Adsorption kinetics of MB by hydrogels is influenced by many factors. The influence of initial MB concentration, time, pH, and crosslinking agent on hydrogel adsorption behavior is investigated in this study. The adsorption capacity of S-AA-1 versus pH is shown in Fig. 5a. The data demonstrated that an increase in pH resulted in enhanced adsorption performance. The same trend was observed for other hydrogels (Fig. S1). At low pH values, the majority of the carboxylic anions in PAA were protonated, resulting in the formation of COOH. As a result, electrostatic repulsion between COO^−^ anions vanished, while hydrogen interactions between polymer chains increased. Ultimately, the structure shrank, making it more difficult for MB molecules to penetrate^55^. Furthermore, the interactions between the carboxylate ions generated competition between excess dissolved H^+^ ions and positively-charged dye molecules, resulting in limited adsorption at these pH values^21^. Adsorption is confined to hydrogen bonds between OH or COOH groups of polymer chains and the amine groups of MB at low pH (below the isoelectric point)^55^. PAA carboxyl groups were deprotonated and converted into carboxylate ions with a negative charge at high pH values. Electrostatic repulsions between negatively-charged groups in hydrogel resulted in structure expansion and increased electrostatic interactions between negatively-charged groups of PAA and positively-charged groups in MB^39^. As a result, adsorption increased dramatically. Furthermore, according to the time-dependent adsorption capacity curves, in all cases, adsorption capacity increased versus time, whereas the adsorption rate was initially rapid, then slowed, and finally reached an equilibrium. The availability of active sites explains the rapid adsorption in the early stages. Adsorption efficiency of the adsorbent at various pH levels was investigated in terms of the initial concentration of MB (Figs. 5c and S2). With an increase in the concentration of dye from 2 to 10 mg/L, the efficiency of the adsorbent increased. However, in a solution with a concentration of 20 mg/L, a significant increase compared to the 10 mg/L solution was not observed and reached a constant value. One of the reasons for such behavior could be increased contact between MB molecules and adsorbent, as well as increased driving force to mass transfer^56^. The literature demonstrates consistent trends regarding the impact of pH, dye concentration, and time on the adsorption of MB by starch-based hydrogels^21, 39^. In contrast to previous studies, it was observed that the adsorption capacity in the present study was comparatively lower. The diminished adsorption capacity may be attributed to several factors, including the reduced concentration of the dye solution, the influence of the cross-linking agent, and the dimensions of the pores within the synthesized hydrogel. Comparisons of hydrogel adsorption capacity curves were made at pH = 2.0 and an initial concentration of 2 mg/L to ascertain the impact of crosslinker concentration on adsorption behavior (Fig. 5b). S-AA-1 was found to have a greater absorption capacity than S-AA-3. Reducing the size of the pores by increasing the amount of crosslinking agent made dye penetration into the hydrogel network structure more difficult, which could be the reason of this phenomenon. Ighalo and coworkers also documented comparable findings regarding the impact of mesoporous size on both adsorption capacity and adsorption kinetics^57^.

The adsorption mechanism and its rate prediction were studied by utilizing linearized PFO, PSO, and IPD models to examine kinetics. The PFO model assumes physical adsorption, and hydrogen bonding and electrostatic interactions between the adsorbent and dye are the primary mechanisms that regulate the adsorption process. According to this model, the adsorption capacity is proportional to the availability of active sites^58^. The PSO model is based on chemical adsorption with ionic interactions between the adsorbate and adsorbent, which controls the adsorption rate. In this model, the adsorption rate is proportional to the square of the difference among accessible active sites and occupied active sites at equilibrium^39^. The IPD model depicts the multi-stage absorption process and includes linear areas with varying slopes^59^. The obtained results were used to fit the PFO and PSO models (Figs. S3–S10), and the kinetic data are displayed in Tables 3, S1, S2, and S3. The higher R^2^ of the PFO model compared to the PSO model and the relatively close q_e_ acquired by the PFO model to the experimental q_e_ in the majority of samples at low pH values (2.0 and 4.8) indicated that the adsorption process is completely in line the PFO model at these two pH values. At high pH values (7.4 and 10.0), the difference between the *q*_*e*_ obtained from the PSO model with the experimental *q*_*e*_ increased, whereas the PFO model still provided a better fit to the experimental data.

**Table 3:** Hydrogel adsorption kinetics parameters for an initial MB concentration of 2 mg/L at varying pH values.

Sample	pH	q_e,experimental_ (mg/g)	PFO model			PSO model			
			k_1_ (1/h)	q_e_ (mg/g)	R^2^	k_2_ (g/(mg h))	q_e_ (mg/g)	h (mg/(g h))	R^2^
S-AA-1	2.0	0.26	0.0664	0.25	0.9864	0.4279	0.28	0.0343	0.9942
	4.8	0.39	0.0565	0.36	0.9982	0.2446	0.43	0.0455	0.9953
	7.4	0.90	0.0613	0.85	0.9970	0.1167	0.98	0.1128	0.9915
	10.0	2.01	0.0663	2.07	0.9978	0.0294	2.36	0.1634	0.9852
S-AA-2	2.0	0.20	0.0604	0.18	0.9958	0.5743	0.22	0.0270	0.9958
	4.8	0.35	0.055	0.32	0.9972	0.2545	0.39	0.0387	0.9964
	7.4	0.84	0.0619	0.84	0.9836	0.0974	0.95	0.0873	0.9888
	10.0	1.92	0.0642	2.00	0.9972	0.0271	2.28	0.1416	0.9834
S-AA3	2.0	0.10	0.0569	0.08	0.9783	1.6829	0.10	0.0187	0.9988
	4.8	0.27	0.0585	0.27	0.9807	0.2927	0.30	0.0272	0.9954
	7.4	0.77	0.0585	0.76	0.9959	0.0847	0.89	0.0665	0.9942
	10.0	1.82	0.0579	1.88	0.9966	0.0265	2.18	0.1262	0.9839

Moreover, the IPD model was applied to all the data for a comprehensive description of MB diffusion into the hydrogel (Figs. S11–S14, Tables 4, and S4–S6). All three linear regions for MB adsorption by the synthesized hydrogels had different slopes away from the origin, suggesting that the adsorption is regulated by a multi-step mechanism. The initial step explains the transfer of the dye to the hydrogel's outer surface via diffusion into the boundary layer^60^. In the second step, MB molecules diffuse continuously into the adsorbent sites in the hydrogel pores via intraparticle diffusion. The third step is the equilibrium of the adsorbent, which occurs when the adsorption capacity has been stabilized and the active sites on the inner surface of the pores are completely saturated with dye molecules^21^. At a constant pH, *k*_*3*_, the initial rate of absorption, increased for stages 1 and 2 of the IPD model when the dye solution concentration was increased from 2 to 5 mg/L. *k*_*3*_ of the second stage was greater than *k*_*3*_ of the first stage for all samples, indicating that film diffusion is faster than intra-particle diffusion. The same trend was also observed when studying the effect of pH on the constants obtained from the IPD model. In all hydrogels, with increasing pH from 2.0 to 10.0, *k*_*3*_ of the first step and *k*_*3*_ and *C* of the second step increased. Hydrogel comparisons revealed that sample S-AA-1 has the highest *k*_*3*_ and *C*, while sample S-AA-2 has the lowest *k*_*3*_ and *C*.

**Table 4:** IPD model adsorption kinetics parameters for hydrogels at different pH values for an initial 2 mg/L MB concentration.

Sample	pH	Intraparticle diffusion model							
		First stage			Second stage			Third stage	
		k_3_ ((mg/g)/h)	C (mg/g)	R^2^	k_3_ ((mg/g)/h)	C (mg/g)	R^2^	C	R^2^
S-AA-1	2.0	0.0375	0.0069	0.9769	0.0210	0.0952	0.9834	0.2600	0.9999
	4.8	0.0578	0	0.9990	0.0313	0.1485	0.9738	0.3920	0.9999
	7.4	0.1326	− 0.0094	0.9896	0.0665	0.9831	0.9831	0.8992	0.9999
	10.0	0.2324	− 0.0318	0.9874	0.1437	0.9152	0.9905	2.0128	0.9999
S-AA-2	2.0	0.0301	0.0032	0.9833	0.0154	0.0820	0.9787	0.2000	0.9999
	4.8	0.0534	− 0.0054	0.9945	0.0274	0.1367	0.9579	0.3520	0.9999
	7.4	0.1159	− 0.0212	0.9894	0.0664	0.6650	0.9891	0.8448	0.9999
	10.0	0.2255	− 0.0666	0.9874	0.1500	0.7697	0.9915	1.9168	0.9999
S-AA3	2.0	0.0223	0.0017	0.9897	0.0066	0.0480	0.9947	0.1000	0.9999
	4.8	0.0412	− 0.0061	0.9860	0.0290	0.0530	0.9898	0.2720	0.9999
	7.4	0.1045	− 0.0193	0.9837	0.0665	0.2572	0.9831	0.7700	0.9999
	10.0	0.2039	− 0.0522	0.9954	0.1693	0.5181	0.9812	1.8208	0.9999

### Study of adsorption isotherms

The equilibrium behavior of the adsorbent and adsorbed molecules was characterized by the Langmuir, Freundlich, and Temkin adsorption isotherms (Figs. S15–S17, Table 5). The Langmuir isotherm indicates monolayer adsorption on homogeneous adsorbent sites, and none of the adsorbate species interact with one another^61^. The negative slope of the graph of the experimental data fitted with this isotherm and the low value of R^2^ indicate that the Langmuir isotherm is unsuitable for prepared hydrogels. The Freundlich isotherm is associated with heterogeneous adsorbent surfaces, where adsorption is not uniform. The *n* parameter is the heterogeneity factor, which indicates the nonlinear relationship between concentration and absorption. If *n* = 1, the adsorption process is linear; if *n* < 1, chemical adsorption happens; and if *n* > 1, physical absorption occurs^62^. All samples had *n* values below 1, suggesting that chemical absorption is occurring. It demonstrated the adsorbent's ability to electrostatically interact with MB molecules. The Temkin isotherm is associated with the uniform distribution of energy during the adsorption process, and the interaction between the adsorbent and the adsorbed molecule causes a linear decrease in the adsorption heat for all molecules^63^. The positive value of *b*_*T*_ in the synthesized samples indicated that the adsorption process is exothermic^64^. Furthermore, the high value of *b*_*T*_ in comparison to *K*_*T*_ indicates that the adsorbent and the adsorbed have a strong interaction^20^. In comparison to the other two isotherms, the Freundlich model with the highest R^2^ value was considered to be the best fit for the empirical evidence.

**Table 5:** Data from fitting experimental data on Freundlich and Temkin isotherms.

Samples	pH	Temkin isotherm			Freundlich isotherm		
		b (kJ/mol)	K_T_ (L/g)	R^2^	n	K_F_ (mg/g)	R^2^
S-AA-1	2.0	2.9	691.5	0.9643	0.65	4341.2	0.9999
	4.8	2.2	738.2	0.9207	0.70	3159.3	0.9950
	7.4	1.2	952.3	0.9651	0.79	3332.9	0.9990
	10.0	0.5	1504.0	0.9624	0.76	16,597.4	0.9989
S-AA-2	2.0	3.1	645.9	0.9856	0.58	10,747.2	0.9970
	4.8	2.4	716.5	0.9275	0.68	3573.9	0.9921
	7.4	1.28	934.1	0.9636	0.79	2987.8	0.9986
	10.0	0.58	1445.5	0.9634	0.78	12,093.2	0.9980
S-AA-3	2.0	3.12	565.2	0.9696	0.42	223,596.9	0.9651
	4.8	2.34	647.2	0.9402	0.58	14,077.0	0.9965
	7.4	1.31	890.3	0.9648	0.76	3726.0	0.9994
	10.0	0.61	1384.6	0.9638	0.79	10,279.4	0.9984

### Study of adsorption mechanism

**Figure 6:**
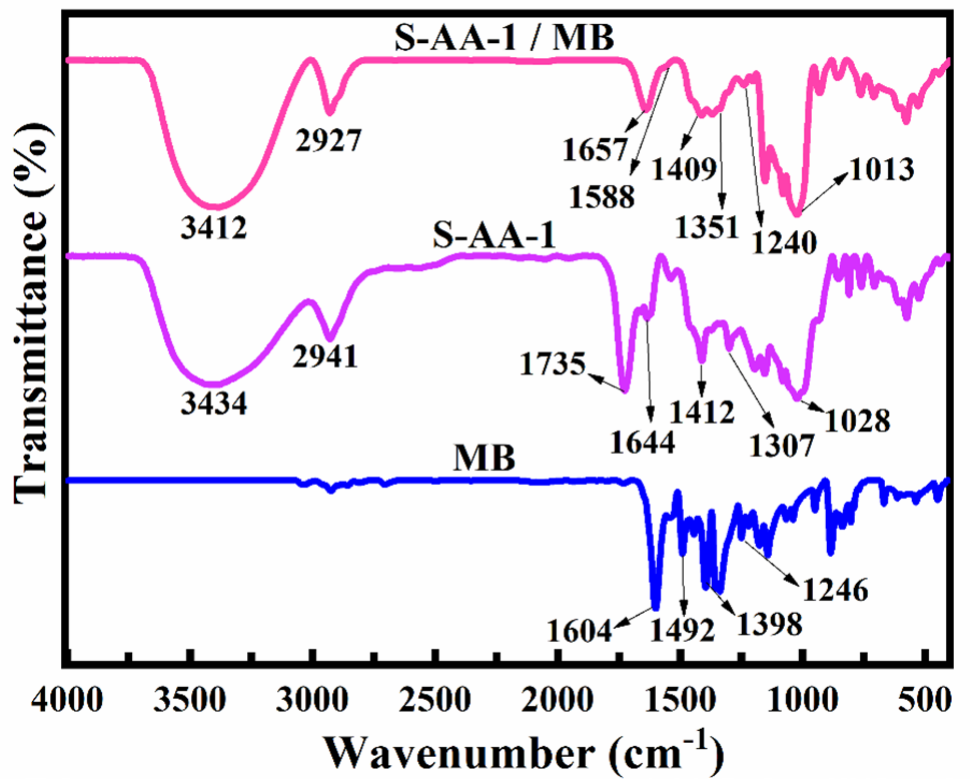
FTIR spectra of MB, S-AA-1 and S-AA-1/MB.

**Figure 7:**
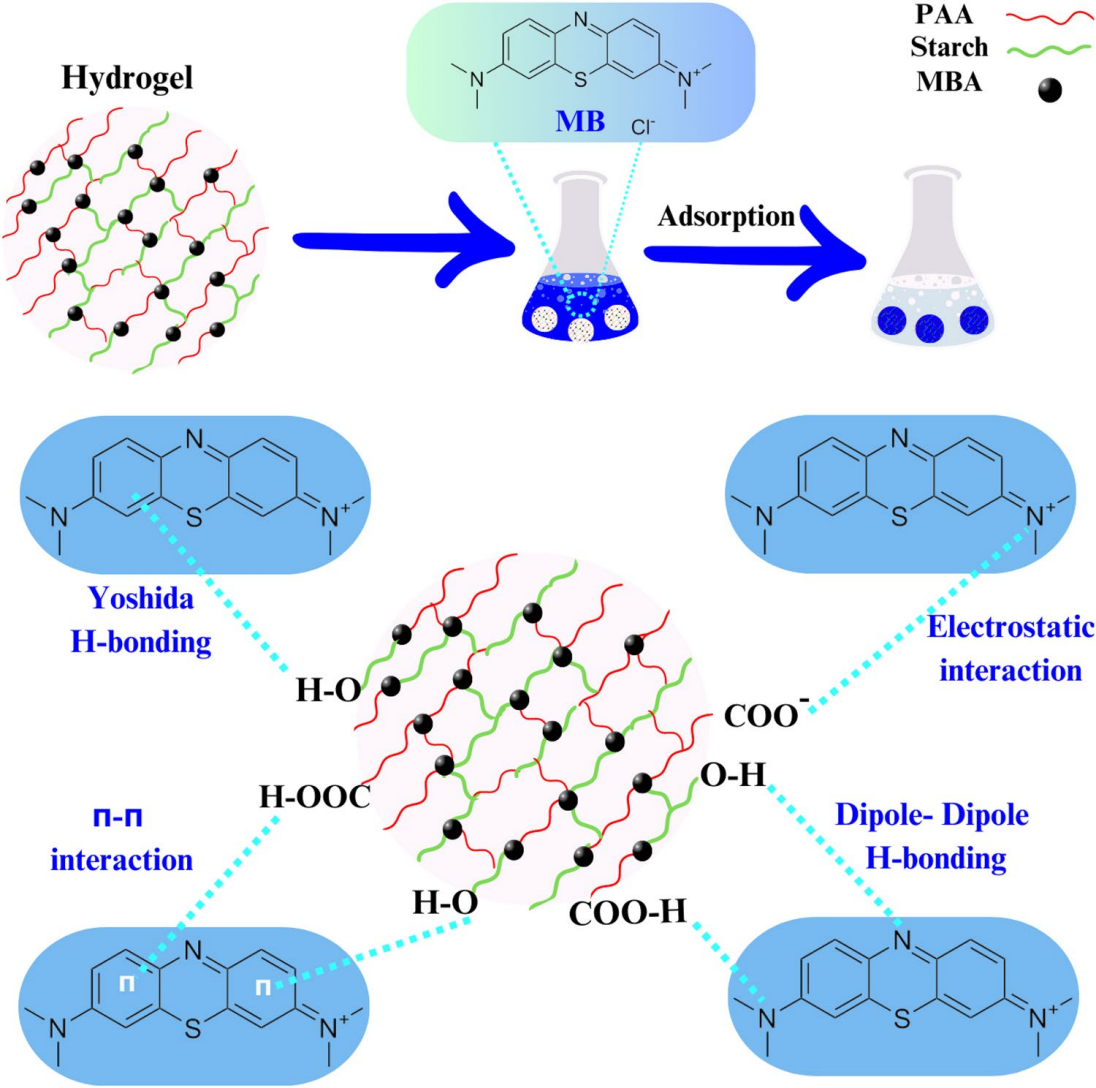
Possible adsorption mechanisms of MB onto the synthesized hydrogel.

One of the challenges encountered in the field of adsorption pertains to the mechanism of adsorption. In order to investigate the adsorption mechanism of MB using hydrogels based on starch/PAA and to ascertain the potential interaction between the adsorbent and adsorbate, FTIR spectroscopy was conducted on MB, S-AA-1 prior to adsorption, and S-AA-1 after adsorption (S-AA-1/MB), as depicted in Fig. 6. The spectral peaks associated with the characterization of MB were observed at wavenumbers of 1604 cm^−1^, 1492 cm^−1^, 1398 cm^−1^, and 1246 cm^−1^. These wavenumbers correspond to the stretching vibration of the aromatic ring, the vibration of the heterocycle skeleton, the symmetric bending vibration of the CH_3_ groups in the dimethylamine groups, and the CN stretching vibration, respectively^65^. In the spectral analysis of S-AA-1/MB, alongside the characteristic peaks associated with hydrogel characterization, distinct peaks indicative of MB identification were observed. It is important to point out that there was a slight shift observed in the peaks. Hence, the adsorption of dye by the synthesized hydrogel was confirmed through FTIR analysis. The findings indicate that the likely mechanism of MB adsorption by the starch/PAA hydrogel involves π-π interaction, dipole–dipole hydrogen bonding, and Yoshida hydrogen bonding^66^. Another potential mechanism for adsorption is the electrostatic interactions between charged groups in the hydrogel and charged groups in MB. This mechanism was verified by investigating the impact of pH on the adsorption capacity. An increase in the number of charged groups led to the generation of a greater number of binding sites within the hydrogel, thereby resulting in an augmented adsorption capacity. Figure 7 illustrates the various mechanisms of MB adsorption on the hydrogel.

## Conclusions

This study involved the synthesis of sustainable porous starch hydrogels that were grafted with PAA. Three different molar ratios of PAA to MBA (95:5, 94:6, and 93:7) were used in the synthesis process. The purpose of this synthesis was to create hydrogels that could effectively adsorb methylene blue from water through solution polymerization. The introduction of PAA through grafting resulted in the disruption of the crystalline arrangement of starch, leading to the formation of a hydrogel with enhanced thermal stability compared to unmodified starch. The augmentation of the cross-linking agent resulted in a reduction in pore size and an increase in the grafting of PAA onto starch. The investigation into the swelling characteristics of the samples revealed an upward trend between pH levels and swelling, as evidenced by an increase in swelling from pH 2.0 to 7.4. Conversely, an inverse relationship was observed between swelling and pH, indicating a decrease in swelling as pH level increased. The hydrogel with a composition ratio of 94:6 exhibited the highest level of swelling, reaching a maximum value of 201.9%, when tested under a pH of 7.4. The modified Power Law model to investigate the mechanism of water transfer to hydrogel showed that Fickian diffusion controls the adsorption mechanism. Furthermore, the swelling kinetics of the hydrogels that were synthesized adhered to Schott's pseudo-second order swelling kinetic model. The study investigated the impact of various parameters, including pH, dye solution concentration, cross-linking agent quantity, and time, on the adsorption behavior of methylene blue by hydrogels. The observed behavior of increased adsorption capacity, resulting from the increase in pH from 2.0 to 10.0 and the initial amount of dye, can be attributed to two factors: the enhancement of electrostatic interactions and the greater availability of active sites for adsorption. The absorption capacity experienced a decrease as a result of the increased cross-linking factor, which led to a more rigid structure and increased resistance to die penetration. The dye solution with a concentration of 20 mg/L at pH 10.0 exhibited the highest adsorption capacity and adsorbent efficiency (26.7 mg/g, 66.7%) when using a hydrogel with a ratio of 95:5. The analysis of hydrogel experimental data using the pseudo-first order kinetic model and the Freundlich isotherm revealed that physisorption takes place in a non-uniform manner on surfaces of heterogeneous adsorbents. Additionally, the Temkin isotherm study demonstrated that the adsorption mechanism of methylene blue by the synthesized hydrogels exhibits an exothermic nature.

## Supplementary Information

Supplementary Information.

## Data Availability

The datasets generated and/or analyzed during the current study are not publicly available at this time as the data form part of an ongoing study. However, the datasets are available from the corresponding author (Mehdi Salami-Kalajahi, m.salami@sut.ac.ir) on reasonable request.
