# Supplementary material for: Adsorption kinetics of methylene blue from wastewater using pH-sensitive starch-based hydrogels
Source: Sci Rep. 2023 Jul 24;13:11900. doi: 10.1038/s41598-023-39241-z (PMC10366085; doi:10.1038/s41598-023-39241-z)
Supplement: Supplementary file 1 — Supplementary Information. [file 41598_2023_39241_MOESM1_ESM.docx]

**Supporting Information for**

**Adsorption kinetics of methylene blue from wastewater using pH-sensitive starch-based hydrogels**

Fatemeh Mohammadzadeh^1^, Marzieh Golshan^2,3^, Vahid Haddadi-Asl^1,^* , Mehdi Salami-Kalajahi^2,3,^*

^1^ Department of Polymer Engineering and Color Technology, Amirkabir University of Technology, Tehran, Iran.

^2^ Faculty of Polymer Engineering, Sahand University of Technology, P.O. Box 51335-1996, Tabriz, Iran

^3^ Institute of Polymeric Materials, Sahand University of Technology, P.O. Box 51335-1996, Tabriz, Iran

* Correspondence concerning this article should be addressed to

Mehdi Salami-Kalajahi : Email : m.salami@sut.ac.ir, Tel. /Fax : +98 41 33459097

Vahid Haddadi-Asl : Email : haddadi@aut.ac.ir, Tel. /Fax : +98 21 64542403

# **S1. Effect of pH on adsorption capacity**


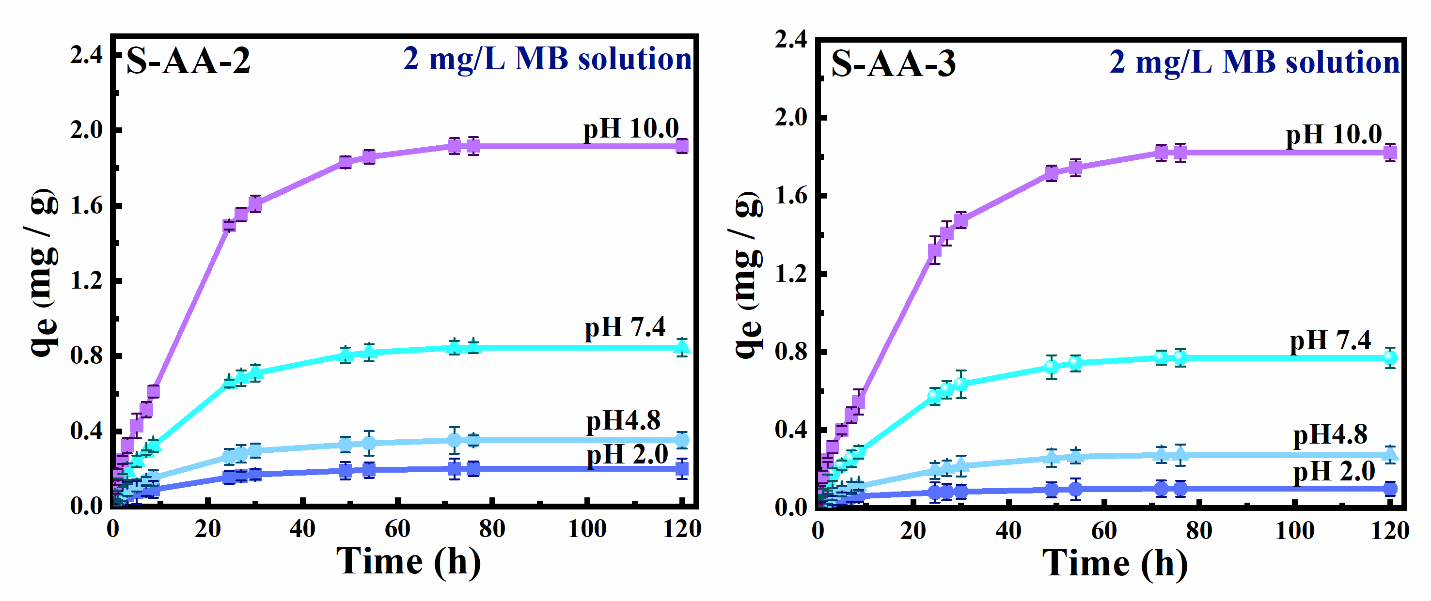


**Figure S1: The effect of pH on the adsorption capacity of S-AA-2 and S-AA-3 for 2 mg/L MB solution**

# **S2. The effect of the initial dye concentration on the adsorption efficiency**


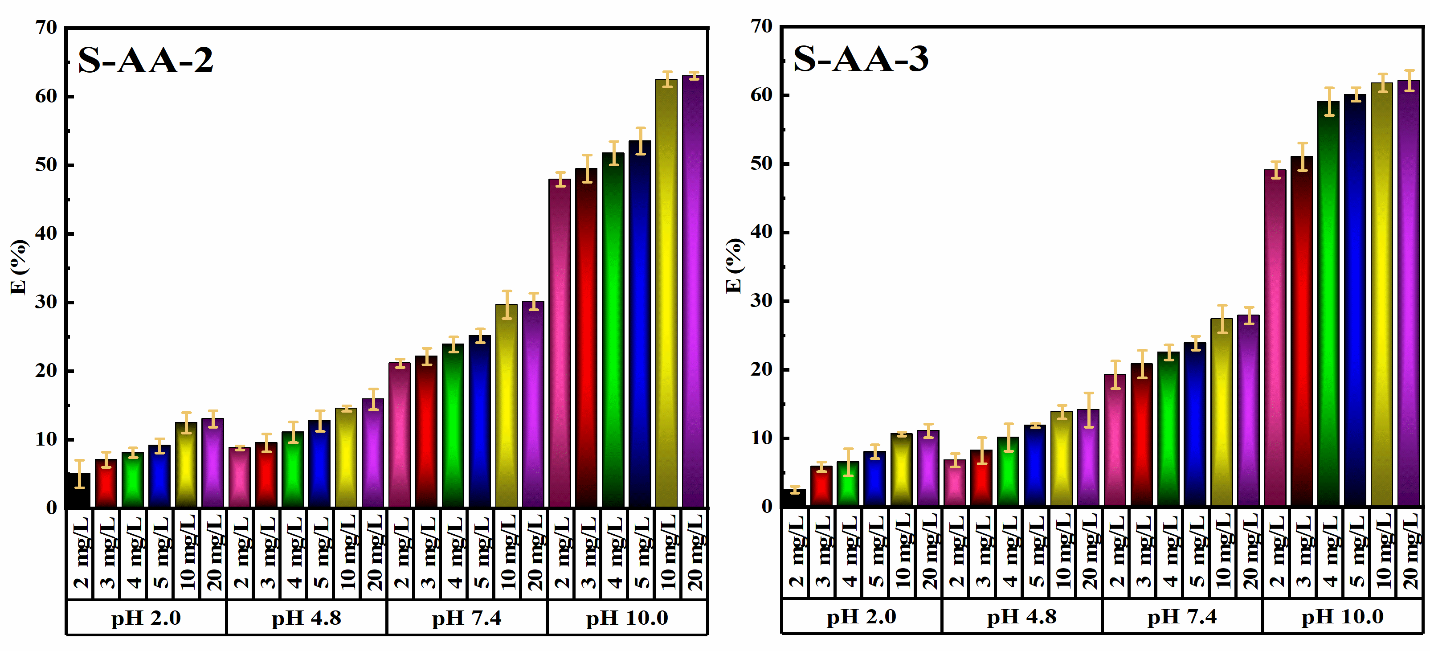


**Figure S2: The effect of MB solution concentration on the adsorption efficiency of S-AA-2 and S-AA-3 at different pH**

# **S3. Fitting the experimental data with different kinetic Models**


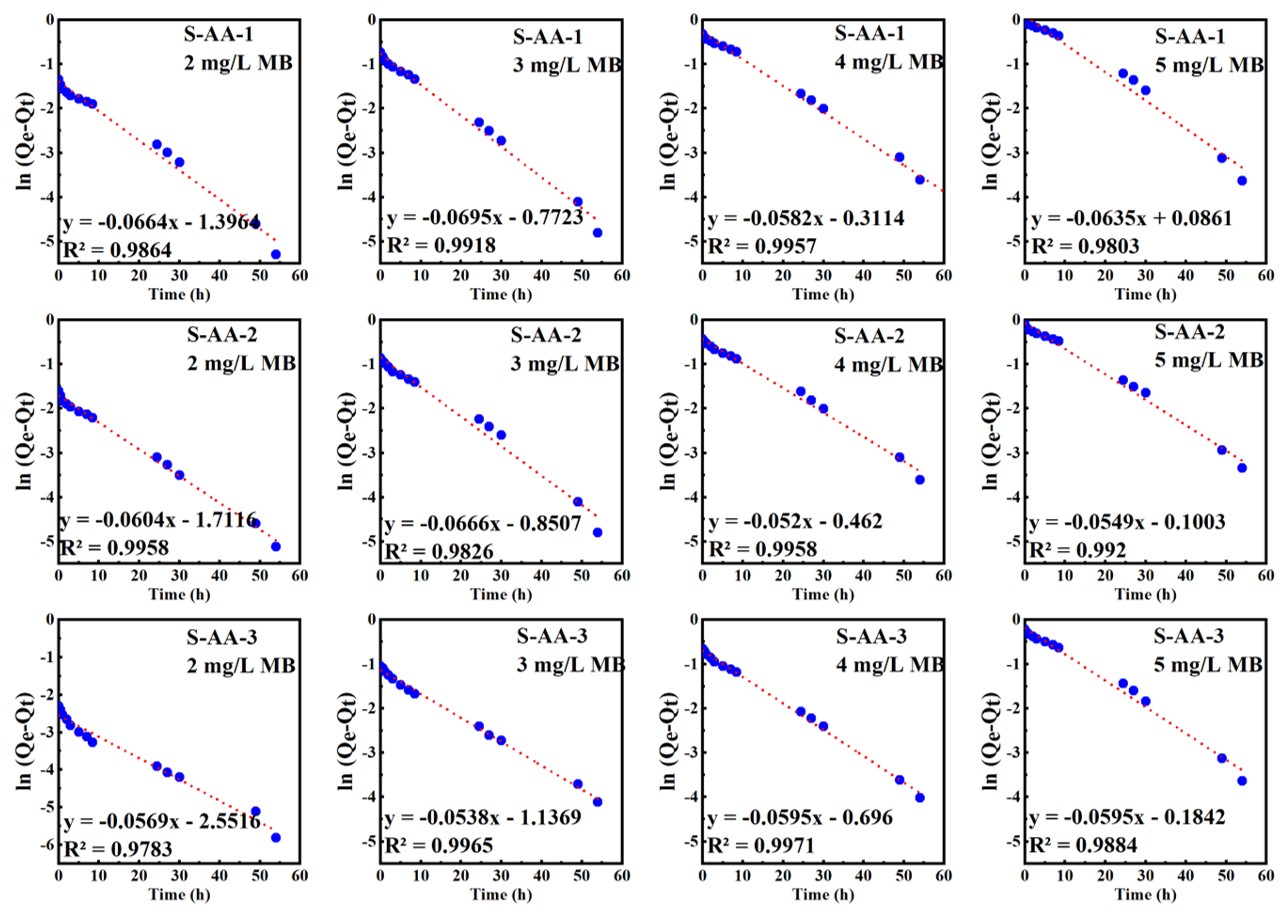


**Figure S3: Fitting the experimental data with the PFO model at pH = 2.0**


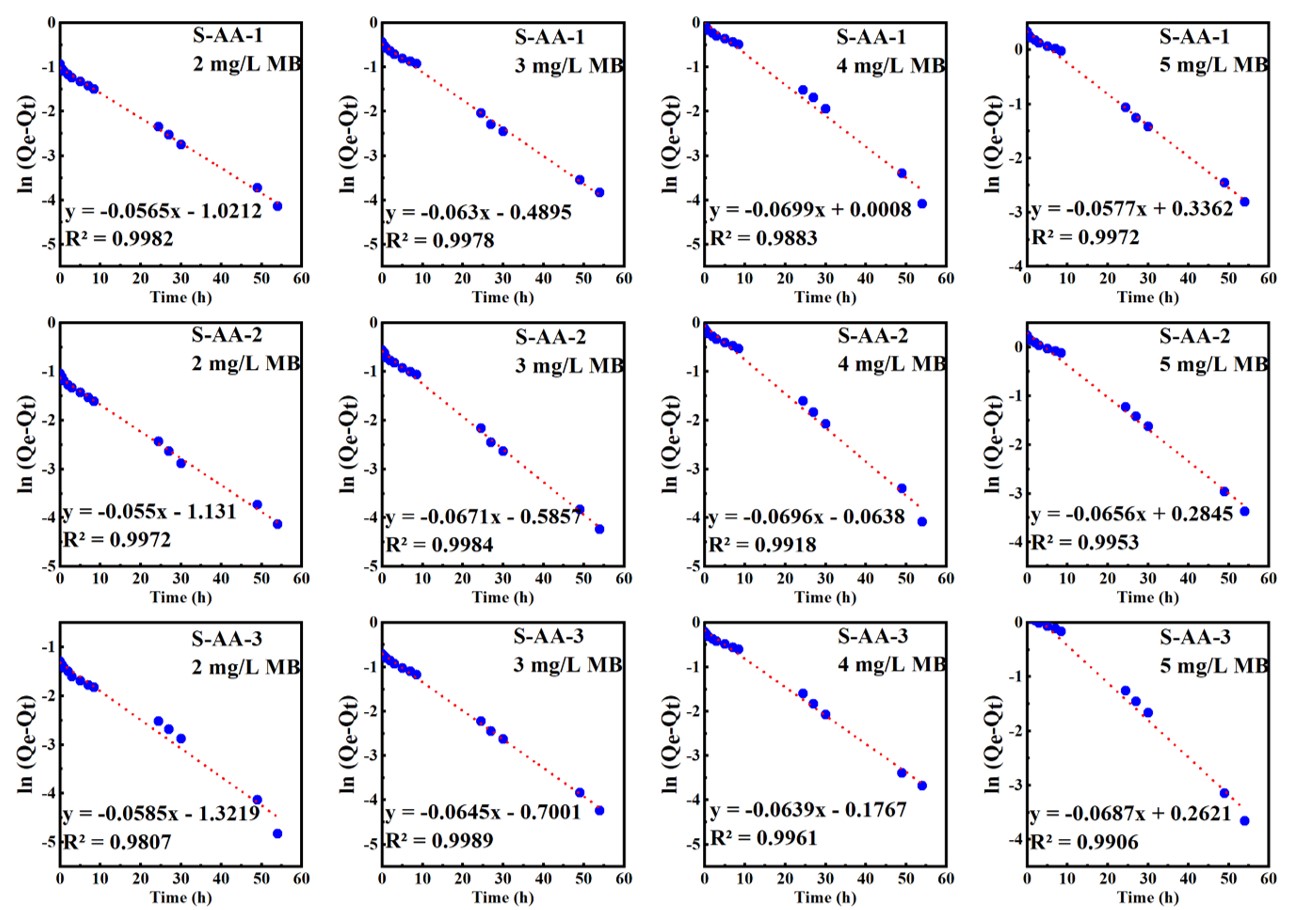


**Figure S4: Fitting the experimental data with the PFO model at pH = 4.8**


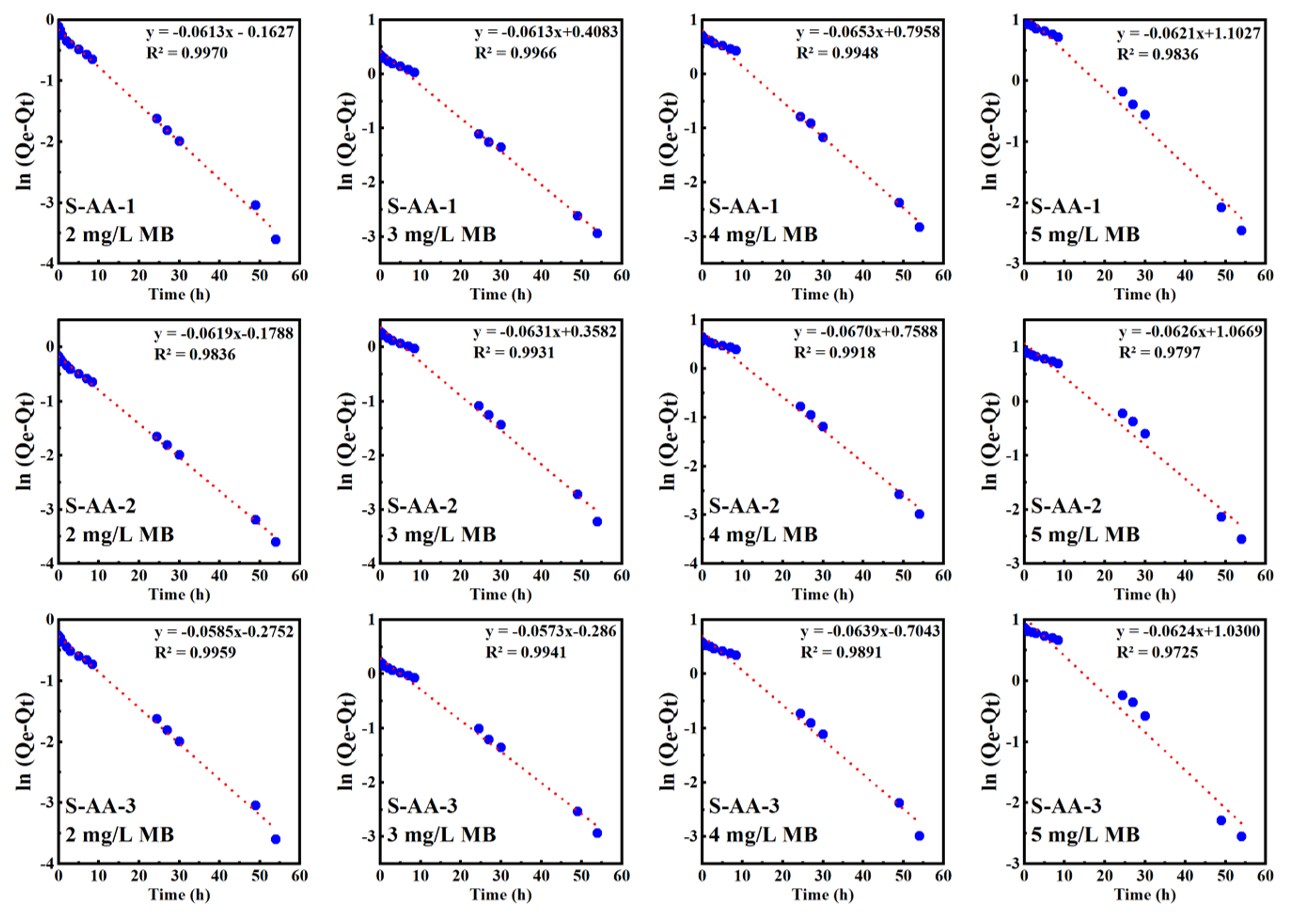


**Figure S5: Fitting the experimental data with the PFO model at pH = 7.4**


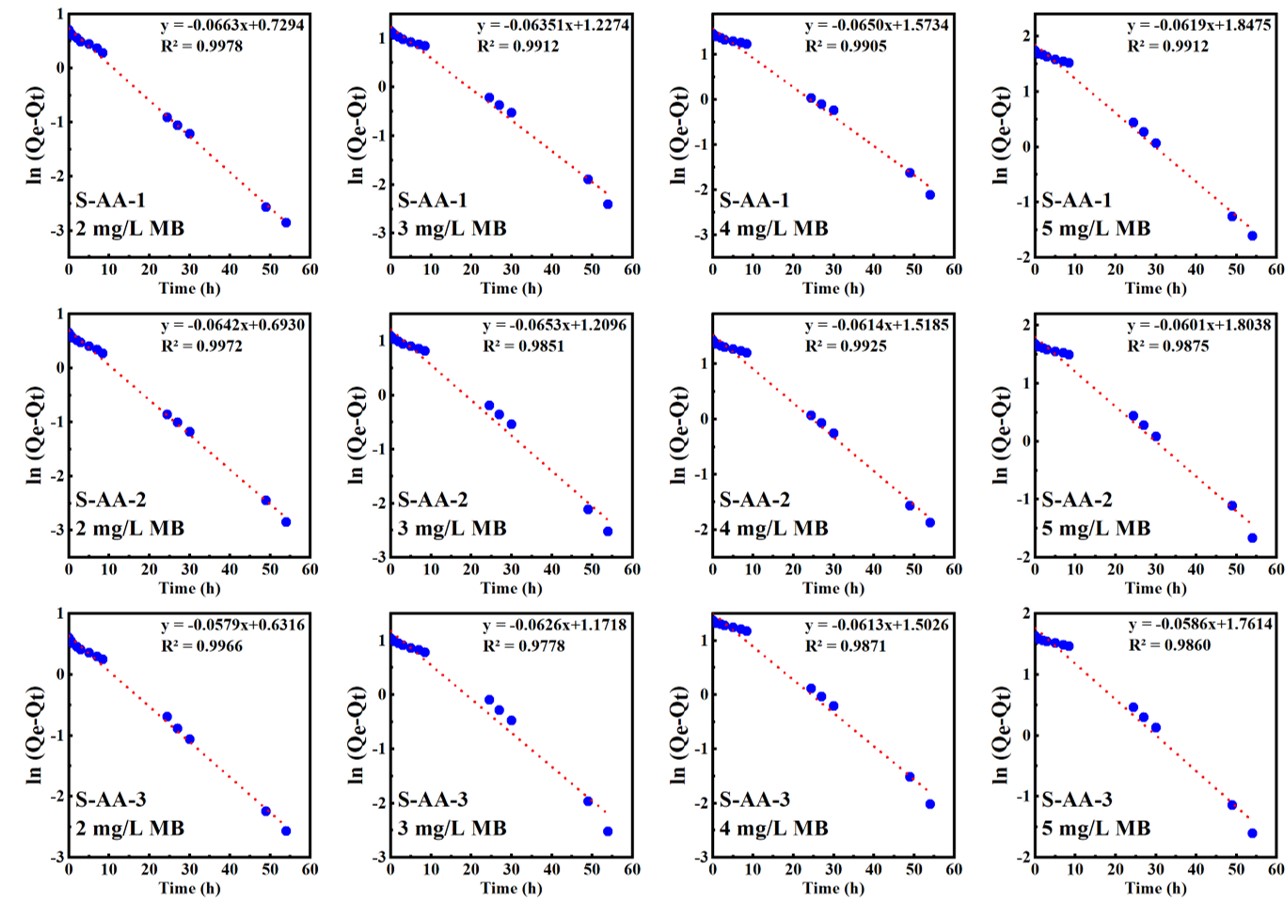


**Figure S6: Fitting the experimental data with the PFO model at pH = 10.0**


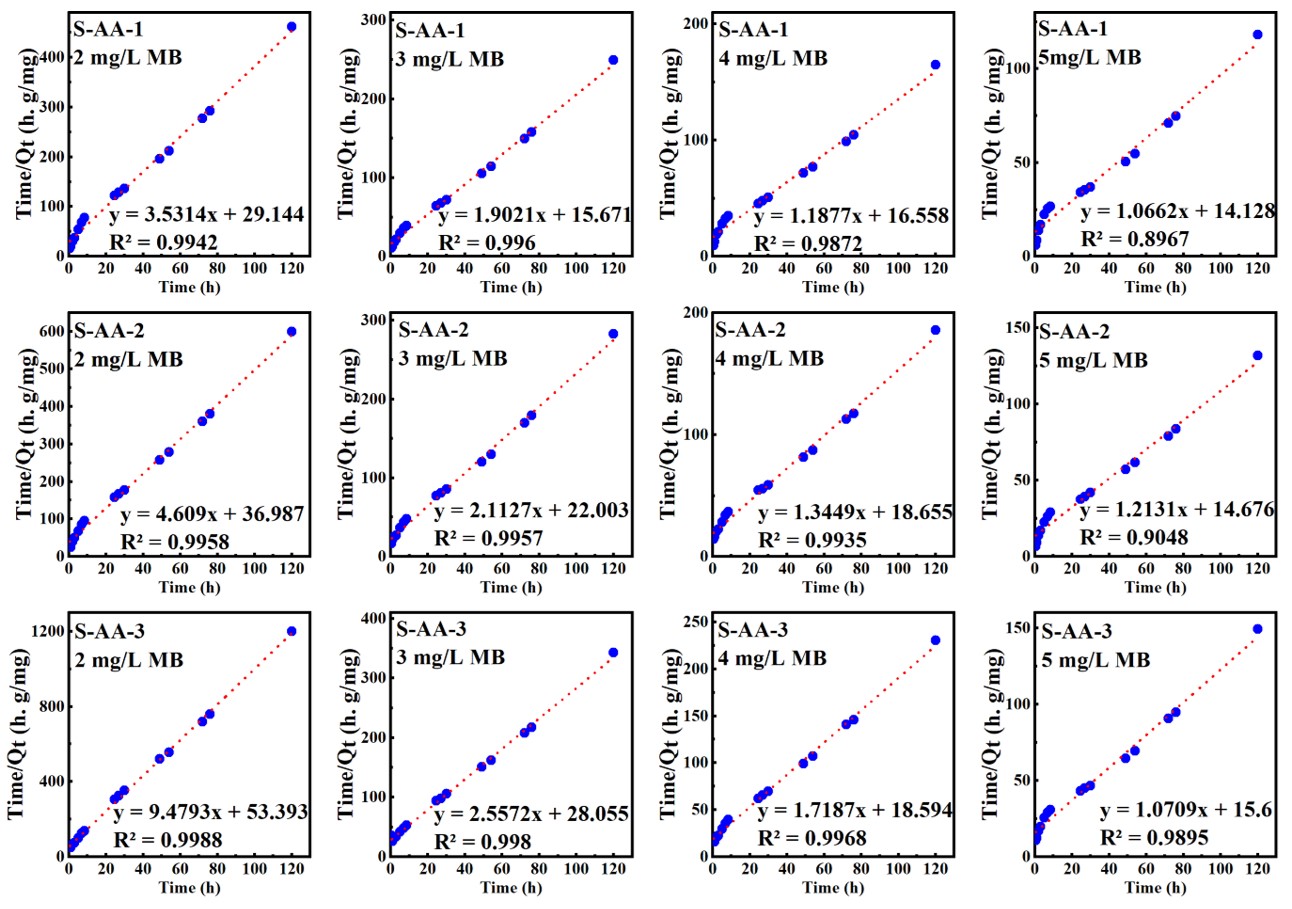


**Figure S7: Fitting the experimental data with the PSO model at pH = 2.0**


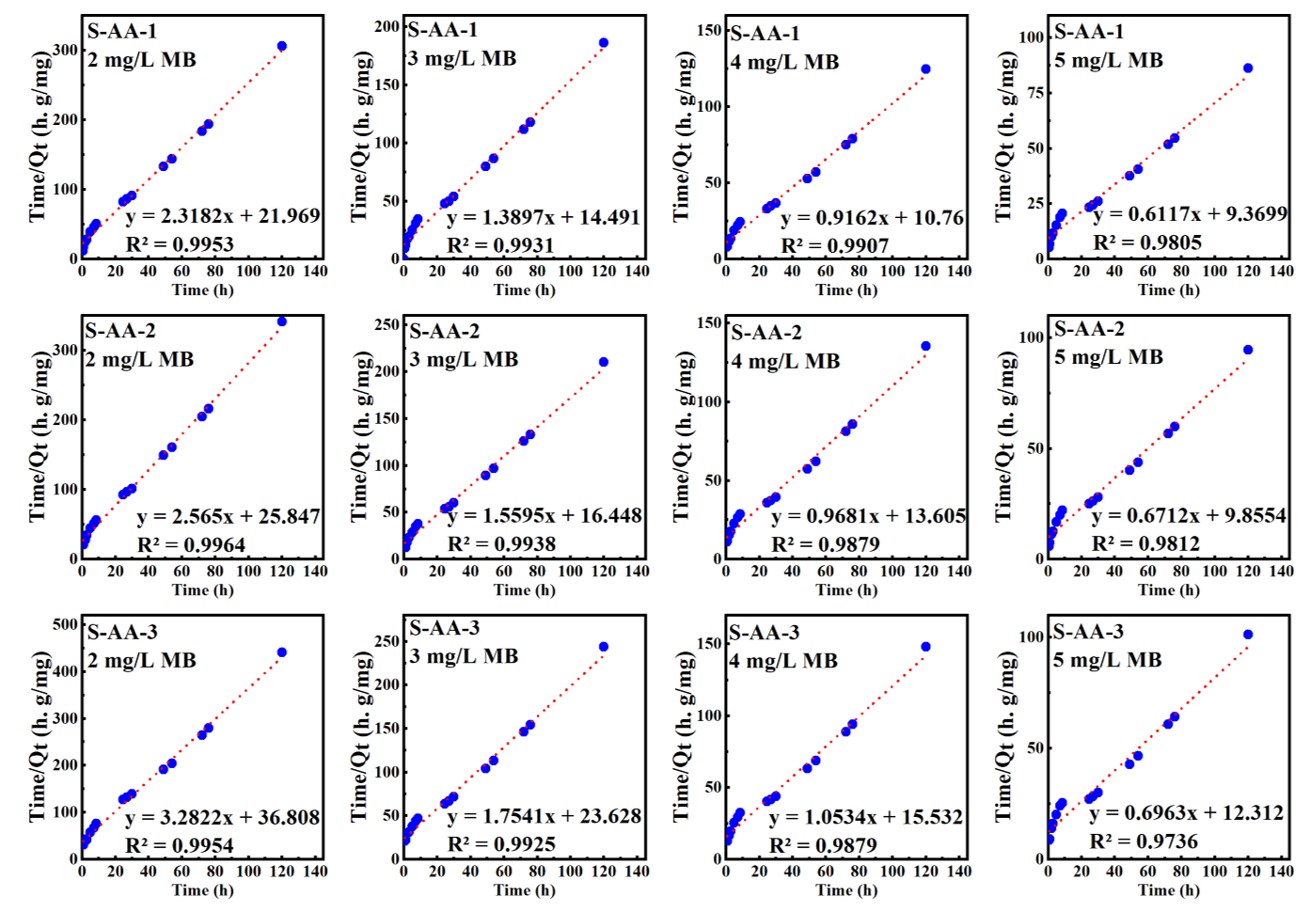


**Figure S8: Fitting the experimental data with the PSO model at pH = 4.8**


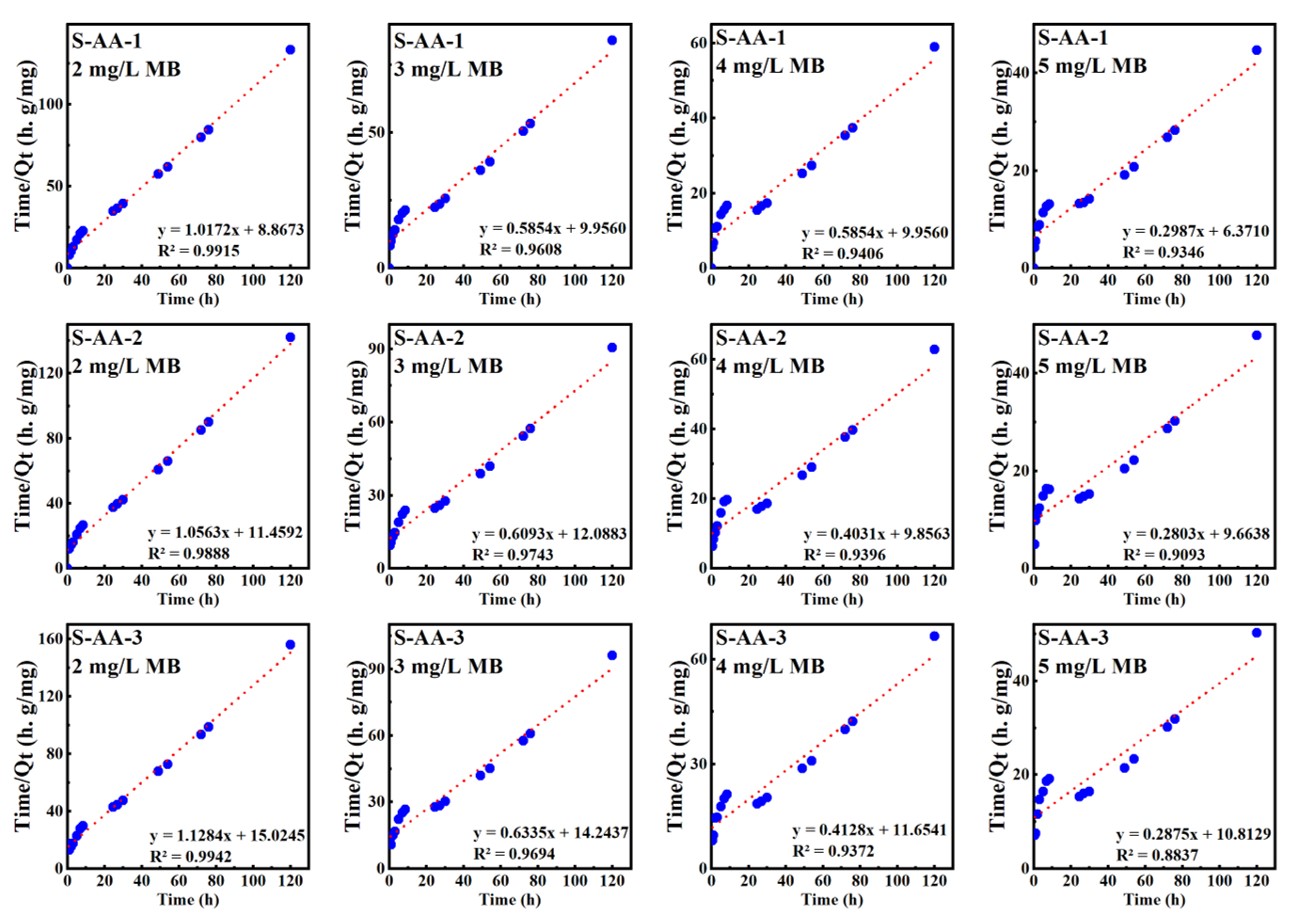


**Figure S9: Fitting the experimental data with the PSO model at pH = 7.4**


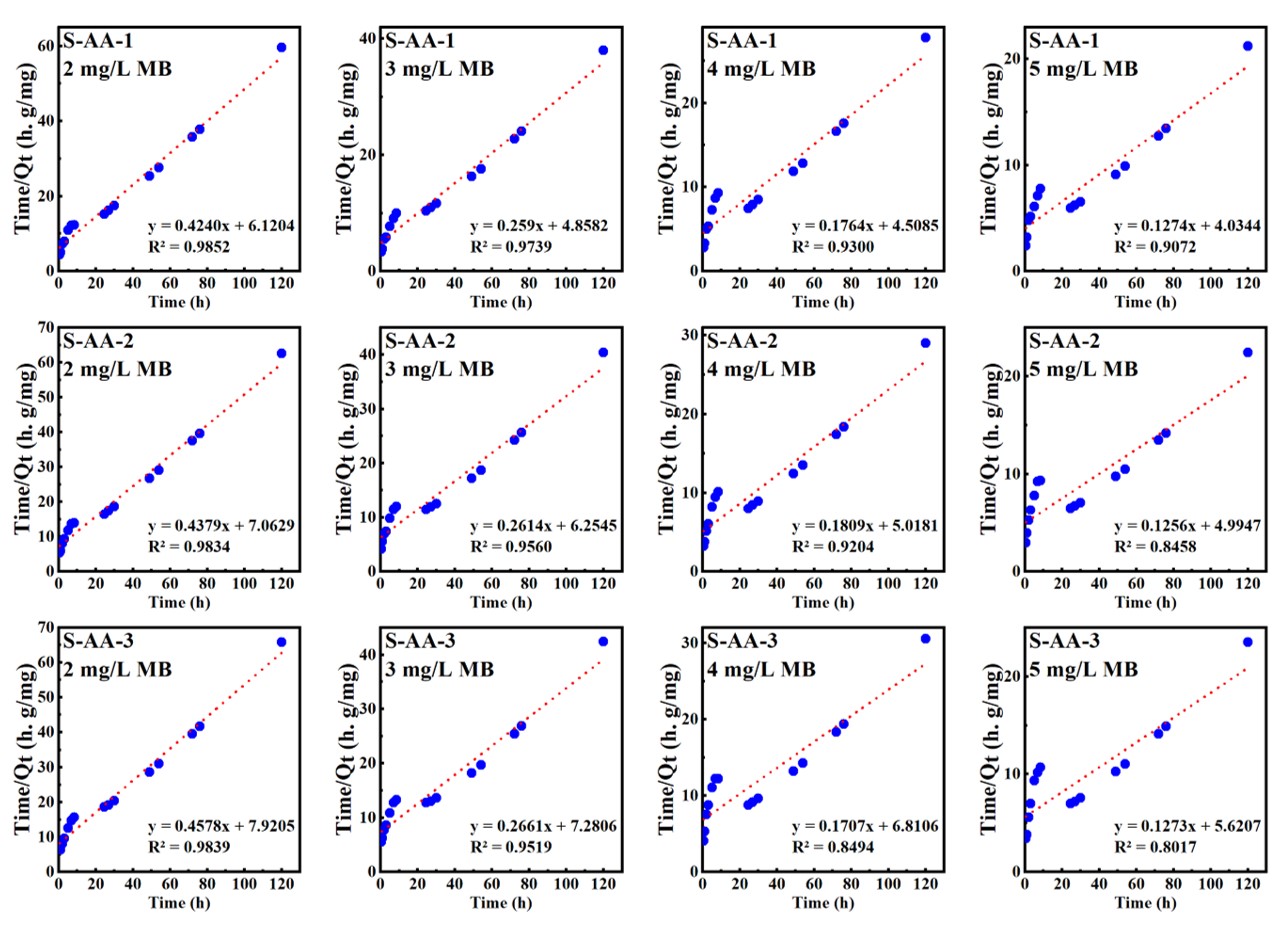


**Figure S10: Fitting the experimental data with the PSO model at pH = 10.0**


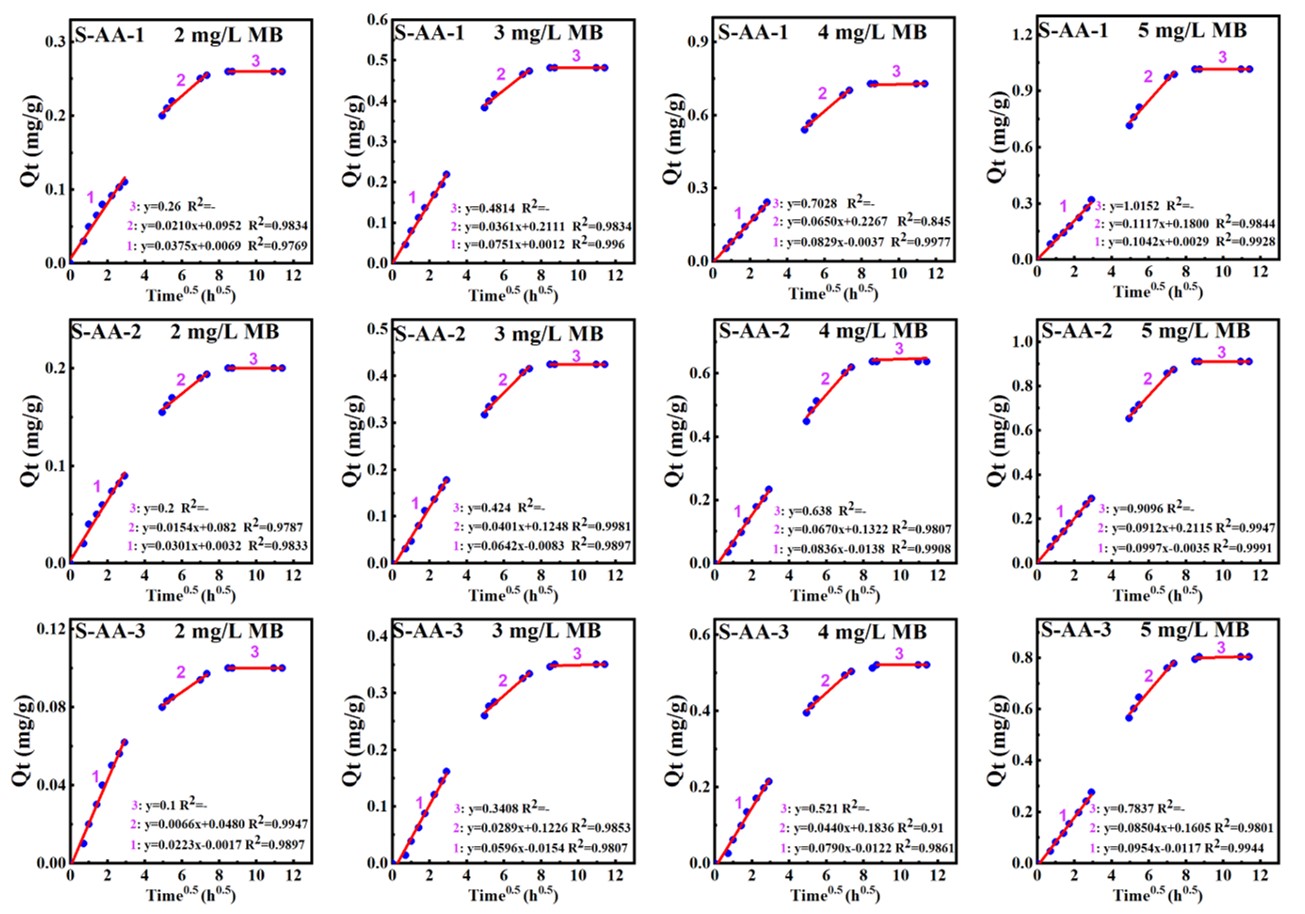


**Figure S11: Fitting the experimental data with the IPD model at pH = 2.0**


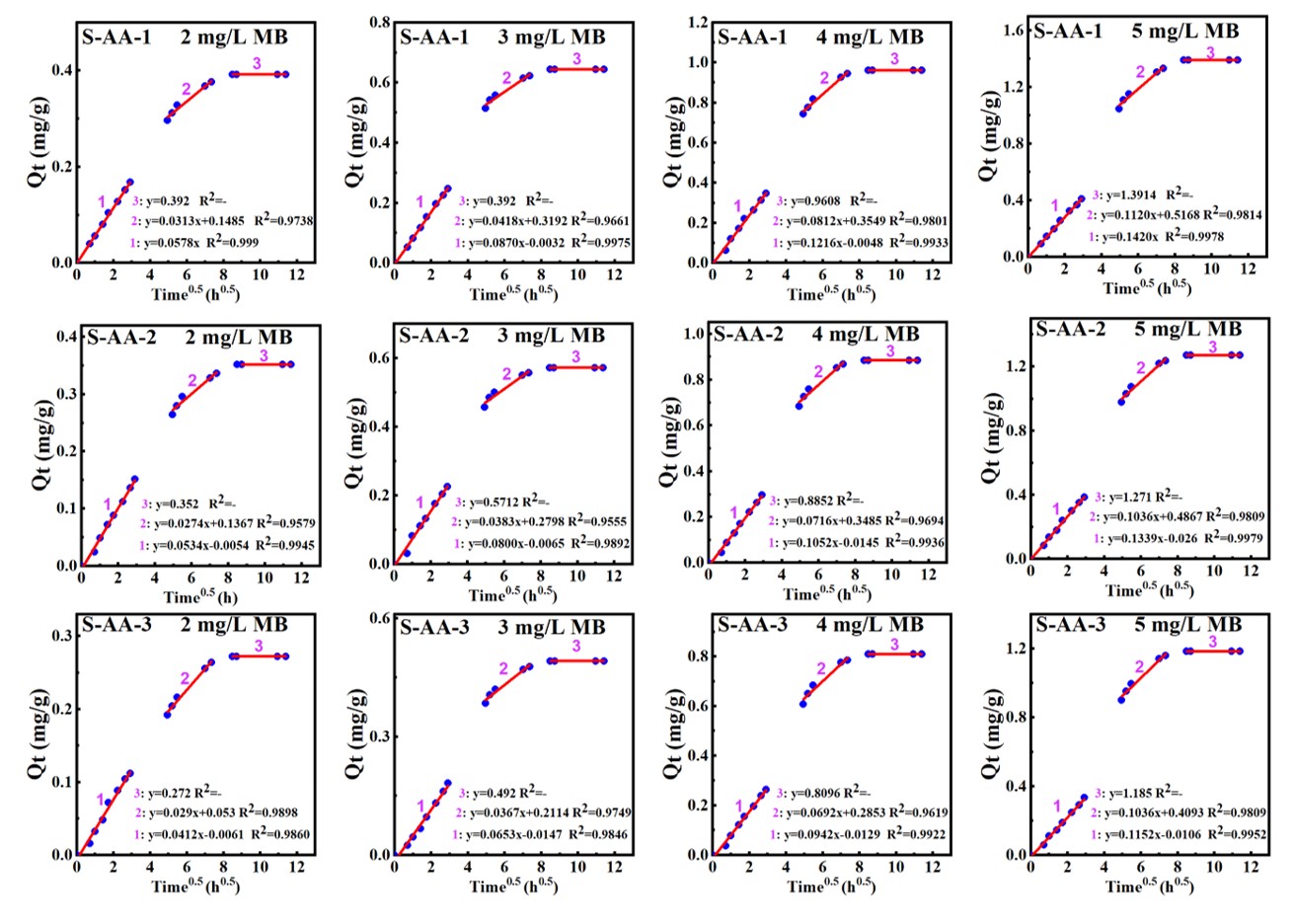


**Figure S12: Fitting the experimental data with the IPD model at pH = 4.8**


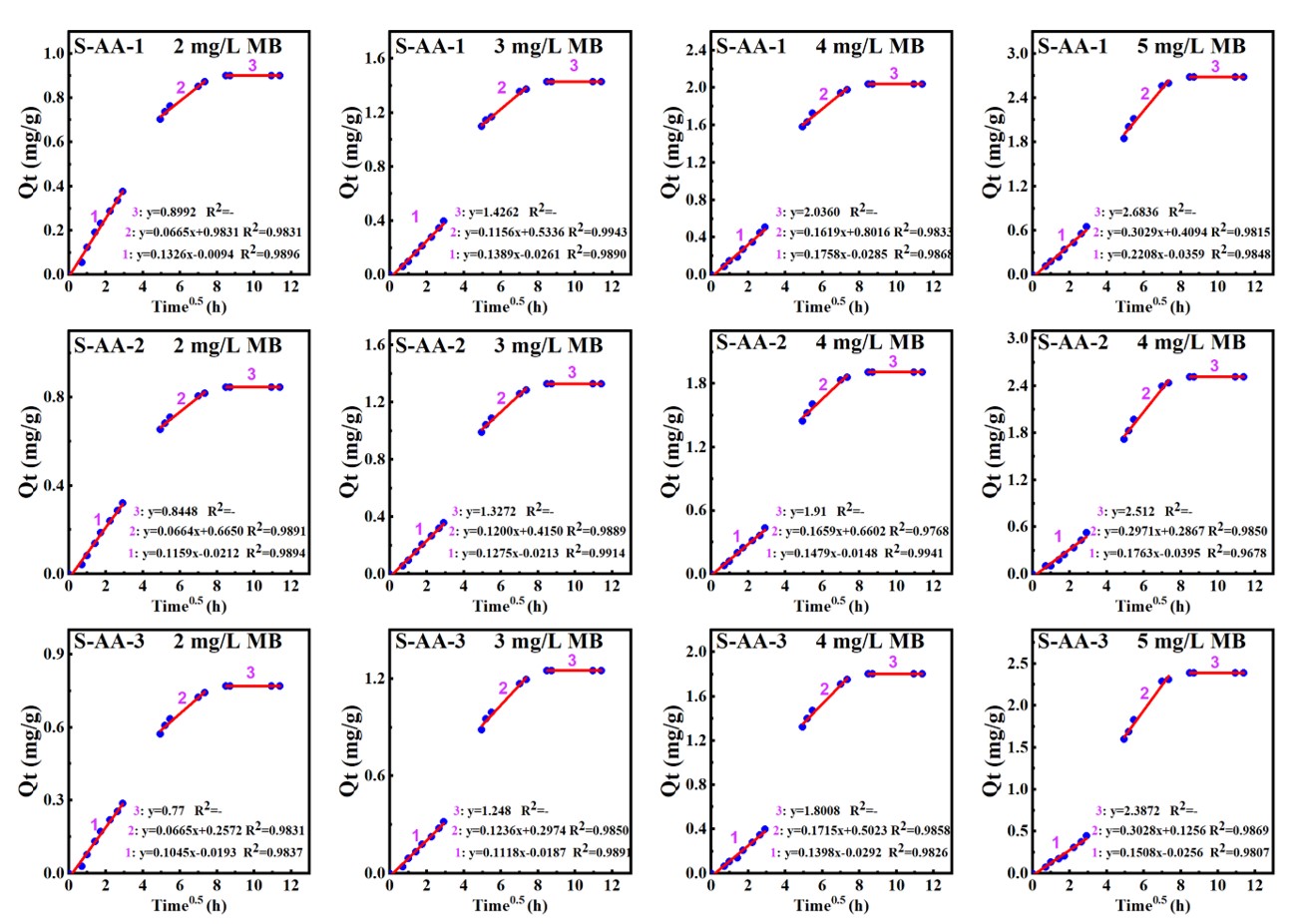


**Figure S13: Fitting the experimental data with the IPD model at pH = 7.4**


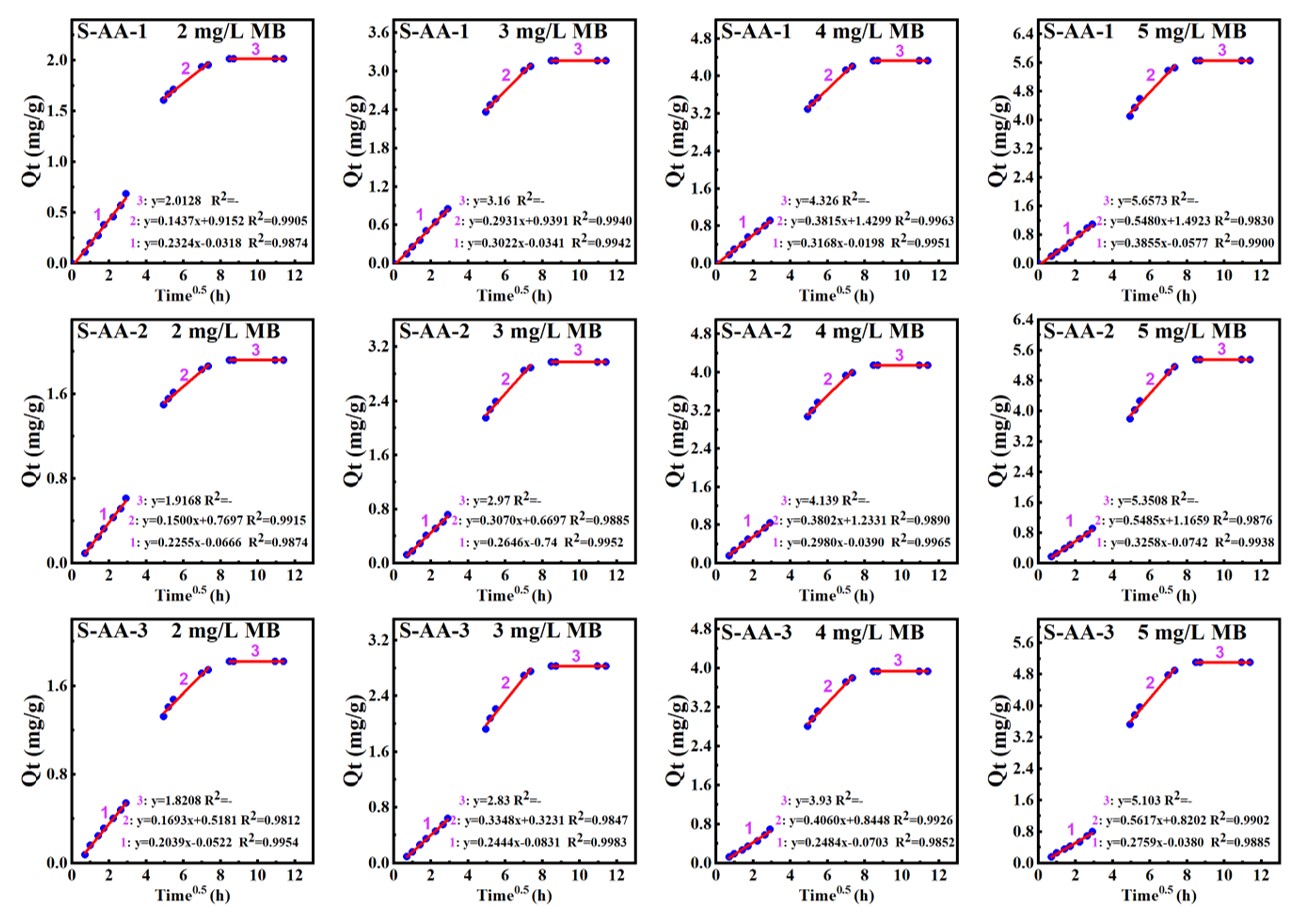


**Figure S14: Fitting the experimental data with the IPD model at pH = 10.0**

# **S4. Extracting data obtained from fitting experimental data with different kinetic models**

**Table S1: Adsorption kinetics parameters of hydrogels at different pH for initial 3 mg/L MB concentration**

| **Sample** | **pH** | **q_e,Experimental_**  **(mg/g)** | **PFO model** | | | **PSO model** | | | |
| --- | --- | --- | --- | --- | --- | --- | --- | --- | --- |
|  |  |  | **k_1_**  **(****1/h)** | **q_e_**  **(mg/g)** | **R^2^** | **k_2_**  **(****g/(mg.h))** | **q_e_**  **(mg/g)** | **h**  **(mg/(g.h))** | **R^2^** |
| **S-AA-1** | 2.0 | 0.48 | 0.0695 | 0.46 | 0.9918 | 0.2309 | 0.52 | 0.0638 | 0.9960 |
|  | 4.8 | 0.64 | 0.063 | 0.61 | 0.9978 | 0.1333 | 0.72 | 0.0690 | 0.9931 |
|  | 7.4 | 1.43 | 0.0613 | 1.50 | 0.9966 | 0.0344 | 1.71 | 0.1004 | 0.9608 |
|  | 10.0 | 3.16 | 0.0653 | 3.41 | 0.9912 | 0.0138 | 3.86 | 0.2058 | 0.9739 |
| **S-AA-2** | 2.0 | 0.42 | 0.0666 | 0.43 | 0.9826 | 0.2028 | 0.47 | 0.0454 | 0.9957 |
|  | 4.8 | 0.57 | 0.0671 | 0.56 | 0.9984 | 0.1479 | 0.64 | 0.0608 | 0.9938 |
|  | 7.4 | 1.33 | 0.0631 | 1.43 | 0.9931 | 0.0307 | 1.64 | 0.0827 | 0.9743 |
|  | 10.0 | 2.97 | 0.0653 | 3.35 | 0.9851 | 0.0109 | 3.82 | 0.1599 | 0.9560 |
| **S-AA3** | 2.0 | 0.35 | 0.0538 | 0.32 | 0.9965 | 0.2331 | 0.39 | 0.036 | 0.9980 |
|  | 4.8 | 0.49 | 0.0645 | 0.50 | 0.9989 | 0.1302 | 0.57 | 0.0423 | 0.9925 |
|  | 7.4 | 1.25 | 0.0573 | 0.75 | 0.9941 | 0.0281 | 1.58 | 0.0702 | 0.9694 |
|  | 10.0 | 2.83 | 0.0626 | 3.23 | 0.9778 | 0.0097 | 3.76 | 0.1373 | 0.9519 |

**Table S2: Adsorption kinetics parameters of hydrogels at different pH for initial 4 mg/L MB concentration**

| **Sample** | **pH** | **q_e,Experimental_**  **(mg/g)** | **PFO model** | | | **PSO model** | | | |
| --- | --- | --- | --- | --- | --- | --- | --- | --- | --- |
|  |  |  | **k_1_**  **(1/h)** | **q_e_**  **(mg/g)** | **R^2^** | **k_2_**  **(g/(mg.h))** | **q_e_**  **(mg/g)** | **h**  **(mg/(g.h))** | **R^2^** |
| **S-AA-1** | 2.0 | 0.73 | 0.0582 | 0.73 | 0.9957 | 0.0852 | 0.84 | 0.0604 | 0.9872 |
|  | 4.8 | 0.96 | 0.0699 | 1.00 | 0.9883 | 0.0780 | 1.09 | 0.0929 | 0.9907 |
|  | 7.4 | 2.04 | 0.0653 | 2.21 | 0.9948 | 0.0344 | 1.71 | 0.1004 | 0.9406 |
|  | 10.0 | 4.33 | 0.0650 | 4.82 | 0.9905 | 0.0069 | 5.67 | 0.2218 | 0.9300 |
| **S-AA-2** | 2.0 | 0.65 | 0.052 | 0.63 | 0.9958 | 0.0969 | 0.74 | 0.0536 | 0.9935 |
|  | 4.8 | 0.88 | 0.0696 | 0.94 | 0.9918 | 0.0689 | 1.03 | 0.0735 | 0.9879 |
|  | 7.4 | 1.91 | 0.0670 | 2.13 | 0.9918 | 0.016 | 2.48 | 0.1014 | 0.9396 |
|  | 10.0 | 4.14 | 0.0614 | 4.56 | 0.9925 | 0.006 | 5.53 | 0.1993 | 0.9204 |
| **S-AA3** | 2.0 | 0.52 | 0.0595 | 0.50 | 0.9971 | 0.1589 | 0.58 | 0.0538 | 0.9968 |
|  | 4.8 | 0.81 | 0.0639 | 0.84 | 0.9961 | 0.0714 | 0.95 | 0.0643 | 0.9879 |
|  | 7.4 | 1.80 | 0.0639 | 0.49 | 0.9891 | 0.0146 | 2.42 | 0.086 | 0.9372 |
|  | 10.0 | 3.93 | 0.0613 | 4.49 | 0.9871 | 0.0043 | 5.86 | 0.1468 | 0.8494 |

**Table S3: Adsorption kinetics parameters of hydrogels at different pH for initial 5 mg/L MB concentration**

| **Sample** | **pH** | **q_e,Experimental_**  **(mg/g)** | **PFO model** | | | **PSO model** | | | |
| --- | --- | --- | --- | --- | --- | --- | --- | --- | --- |
|  |  |  | **k_1_**  **(1/h)** | **q_e_**  **(mg/g)** | **R^2^** | **k_2_**  **(g/(mg.h))** | **q_e_**  **(mg/g)** | **h**  **(mg/(g.h))** | **R^2^** |
| **S-AA-1** | 2.0 | 1.01 | 0.0635 | 1.09 | 0.9803 | 0.0805 | 0.94 | 0.0708 | 0.8967 |
|  | 4.8 | 1.39 | 0.0577 | 1.40 | 0.9972 | 0.0400 | 1.63 | 0.1067 | 0.9805 |
|  | 7.4 | 2.68 | 0.0621 | 3.01 | 0.9836 | 0.0140 | 3.35 | 0.1570 | 0.9346 |
|  | 10.0 | 5.66 | 0.0619 | 6.34 | 0.9912 | 0.0040 | 7.85 | 0.2479 | 0.9072 |
| **S-AA-2** | 2.0 | 0.91 | 0.0549 | 0.9046 | 0.9920 | 0.1003 | 0.82 | 0.0681 | 0.9048 |
|  | 4.8 | 1.27 | 0.0656 | 1.33 | 0.9953 | 0.0457 | 1.49 | 0.1015 | 0.9812 |
|  | 7.4 | 2.51 | 0.0626 | 2.91 | 0.9797 | 0.0081 | 3.57 | 0.1035 | 0.9093 |
|  | 10.0 | 5.35 | 0.0601 | 6.07 | 0.9875 | 0.0031 | 7.96 | 0.2002 | 0.8458 |
| **S-AA3** | 2.0 | 0.80 | 0.0595 | 0.83 | 0.9884 | 0.0735 | 0.93 | 0.064 | 0.9895 |
|  | 4.8 | 1.18 | 0.0687 | 1.30 | 0.9906 | 0.0394 | 1.44 | 0.0812 | 0.9736 |
|  | 7.4 | 2.39 | 0.0624 | 2.8 | 0.9725 | 0.0076 | 3.48 | 0.0925 | 0.8837 |
|  | 10.0 | 5.10 | 0.0586 | 5.82 | 0.9860 | 0.0029 | 7.85 | 0.1779 | 0.8017 |

**Table S4: Adsorption kinetics parameters of intraparticle diffusion model for hydrogels at different pH for initial 3 mg/L MB concentration**

| **Sample** | **pH** | **Intraparticle diffusion model** | | | | | | | |
| --- | --- | --- | --- | --- | --- | --- | --- | --- | --- |
|  |  | **First stage** | | | **Second stage** | | | **Third stage** | |
|  |  | **k_3_**  **((mg/g)/h)** | **C**  **(mg/g)** | **R^2^** | **k_3_**  **((mg/g)/h)** | **C**  **(mg/g)** | **R^2^** | **C** | **R^2^** |
| **S-AA-1** | 2.0 | 0.0751 | 0.0012 | 0.9960 | 0.0361 | 0.2111 | 0.9834 | 0.4814 | 0.9999 |
|  | 4.8 | 0.0870 | 0.0032 | 0.9975 | 0.0418 | 0.3192 | 0.9661 | 0.392 | 0.9999 |
|  | 7.4 | 0.1389 | -0.0261 | 0.9890 | 0.1156 | 0.5336 | 0.9943 | 1.4262 | 0.9999 |
|  | 10.0 | 0.3022 | -0.0341 | 0.9942 | 0.2931 | 0.9391 | 0.9940 | 3.1600 | 0.9999 |
| **S-AA-2** | 2.0 | 0.0642 | 0.0083 | 0.9897 | 0.0401 | 0.1248 | 0.9981 | 0.4240 | 0.9999 |
|  | 4.8 | 0.0800 | 0.0065 | 0.9892 | 0.0383 | 0.2798 | 0.9555 | 0.5712 | 0.9999 |
|  | 7.4 | 0.1275 | -0.2130 | 0.9914 | 0.1200 | 0.4150 | 0.9889 | 1.3272 | 0.9999 |
|  | 10.0 | 0.2646 | -0.7400 | 0.9952 | 0.3070 | 0.6697 | 0.9885 | 2.9700 | 0.9999 |
| **S-AA3** | 2.0 | 0.0596 | 0.0154 | 0.9807 | 0.0289 | 0.1226 | 0.9853 | 0.3408 | 0.9999 |
|  | 4.8 | 0.0653 | -0.0147 | 0.9846 | 0.0367 | 0.2114 | 0.9749 | 0.4920 | 0.9999 |
|  | 7.4 | 0.1118 | -0.0187 | 0.9891 | 0.1236 | 0.2974 | 0.9850 | 1.2480 | 0.9999 |
|  | 10.0 | 0.2444 | -0.0083 | 0.9983 | 0.3348 | 0.3231 | 0.9847 | 2.8300 | 0.9999 |

**Table S5: Adsorption kinetics parameters of intraparticle diffusion model for hydrogels at different pH for initial 4 mg/L MB concentration**

| **Sample** | **pH** | **Intraparticle diffusion model** | | | | | | | |
| --- | --- | --- | --- | --- | --- | --- | --- | --- | --- |
|  |  | **First stage** | | | **Second stage** | | | **Third stage** | |
|  |  | **k_3_**  **((mg/g)/h)** | **C**  **(mg/g)** | **R^2^** | **k_3_**  **((mg/g)/h)** | **C**  **(mg/g)** | **R^2^** | **C** | **R^2^** |
| **S-AA-1** | 2.0 | 0.0829 | -0.0037 | 0.9977 | 0.0650 | 0.2267 | 0.8450 | 0.7028 | 0.9999 |
|  | 4.8 | 0.1216 | -0.0048 | 0.9933 | 0.0812 | 0.3549 | 0.9801 | 0.9608 | 0.9999 |
|  | 7.4 | 0.1758 | -0.0285 | 0.9868 | 0.1619 | 0.8016 | 0.9833 | 2.0360 | 0.9999 |
|  | 10.0 | 0.3168 | -0.0198 | 0.9951 | 0.3815 | 1.4299 | 0.9963 | 4.3260 | 0.9999 |
| **S-AA-2** | 2.0 | 0.0836 | -0.0138 | 0.9908 | 0.0670 | 0.1322 | 0.9807 | 0.6380 | 0.9999 |
|  | 4.8 | 0.1052 | -0.0145 | 0.9936 | 0.0716 | 0.3485 | 0.9694 | 0.8852 | 0.9999 |
|  | 7.4 | 0.1479 | -0.0148 | 0.9941 | 0.1659 | 0.6602 | 0.9768 | 1.9100 | 0.9999 |
|  | 10.0 | 0.2980 | -0.0390 | 0.9965 | 0.3802 | 1.2331 | 0.9890 | 4.1390 | 0.9999 |
| **S-AA3** | 2.0 | 0.0790 | -0.0122 | 0.9861 | 0.0440 | 0.1836 | 0.9100 | 0.5210 | 0.9999 |
|  | 4.8 | 0.0942 | -0.0129 | 0.9922 | 0.0692 | 0.2853 | 0.9619 | 0.8096 | 0.9999 |
|  | 7.4 | 0.1398 | -0.0292 | 0.9826 | 0.1715 | 0.5023 | 0.9858 | 1.8008 | 0.9999 |
|  | 10.0 | 0.2484 | -0.0703 | 0.9852 | 0.4060 | 0.8448 | 0.9926 | 3.9300 | 0.9999 |

**Table S6: Adsorption kinetics parameters of intraparticle diffusion model for hydrogels at different pH for initial 5 mg/L MB concentration**

| **Sample** | **pH** | **Intraparticle diffusion model** | | | | | | | |
| --- | --- | --- | --- | --- | --- | --- | --- | --- | --- |
|  |  | **First stage** | | | **Second stage** | | | **Third stage** | |
|  |  | **k_3_**  **((mg/g)/h)** | **C**  **(mg/g)** | **R^2^** | **k_3_**  **((mg/g)/h)** | **C**  **(mg/g)** | **R^2^** | **C** | **R^2^** |
| **S-AA-1** | 2.0 | 0.1042 | 0.0029 | 0.9928 | 0.1117 | 0.1800 | 0.9844 | 1.0152 | 0.9999 |
|  | 4.8 | 0.1420 | 0 | 0.9978 | 0.1120 | 0.5168 | 0.9814 | 1.3914 | 0.9999 |
|  | 7.4 | 0.2208 | -0.0359 | 0.9848 | 0.3029 | 0.4094 | 0.9815 | 2.6836 | 0.9999 |
|  | 10.0 | 0.3855 | -0.0577 | 0.9900 | 0.5480 | 1.4923 | 0.9830 | 5.6573 | 0.9999 |
| **S-AA-2** | 2.0 | 0.0997 | -0.0035 | 0.9991 | 0.0912 | 0.2115 | 0.9947 | 0.9096 | 0.9999 |
|  | 4.8 | 0.1339 | -0.026 | 0.9979 | 0.1036 | 0.4867 | 0.9809 | 1.2710 | 0.9999 |
|  | 7.4 | 0.1763 | -0.0395 | 0.9678 | 0.2971 | 0.2867 | 0.9850 | 2.5120 | 0.9999 |
|  | 10.0 | 0.3258 | -0.0742 | 0.9938 | 0.5485 | 1.1659 | 0.9876 | 5.3508 | 0.9999 |
| **S-AA3** | 2.0 | 0.0954 | -0.0117 | 0.9944 | 0.0850 | 0.1605 | 0.9801 | 0.7837 | 0.9999 |
|  | 4.8 | 0.1152 | -0.0106 | 0.9952 | 0.1036 | 0.4093 | 0.9809 | 1.1850 | 0.9999 |
|  | 7.4 | 0.1508 | -0.0256 | 0.9807 | 0.3028 | 0.1256 | 0.9869 | 2.3872 | 0.9999 |
|  | 10.0 | 0.2759 | -0.0380 | 0.9885 | 0.5617 | 0.8202 | 0.9902 | 5.1030 | 0.9999 |

# **S5. Fitting the experimental data with different isotherm models**


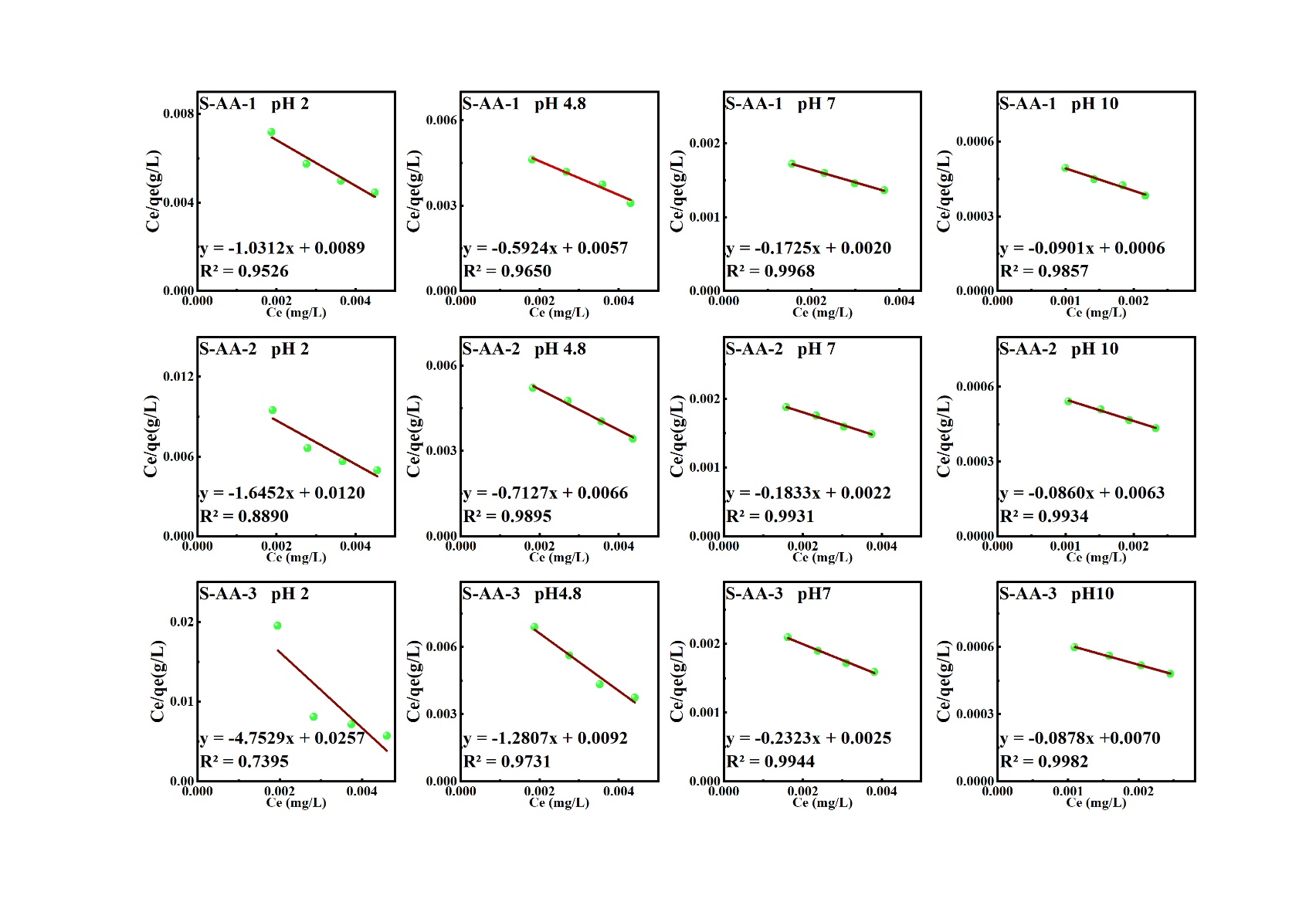


**Figure S15: Fitting the experimental data with the Langmuir isotherm in different pH values**


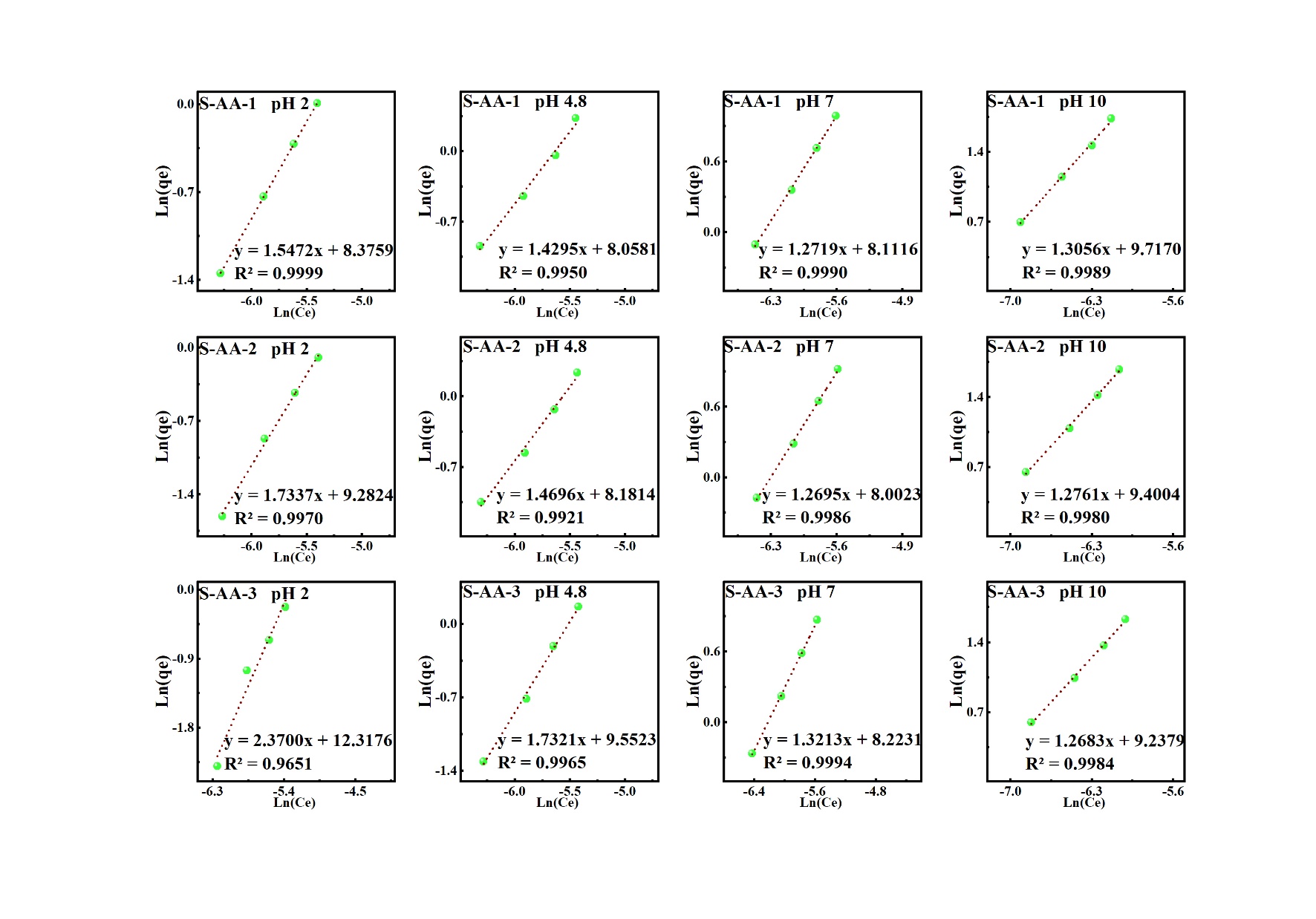


**Figure S16: Fitting the experimental data with the Freundlich isotherm in different pH values**


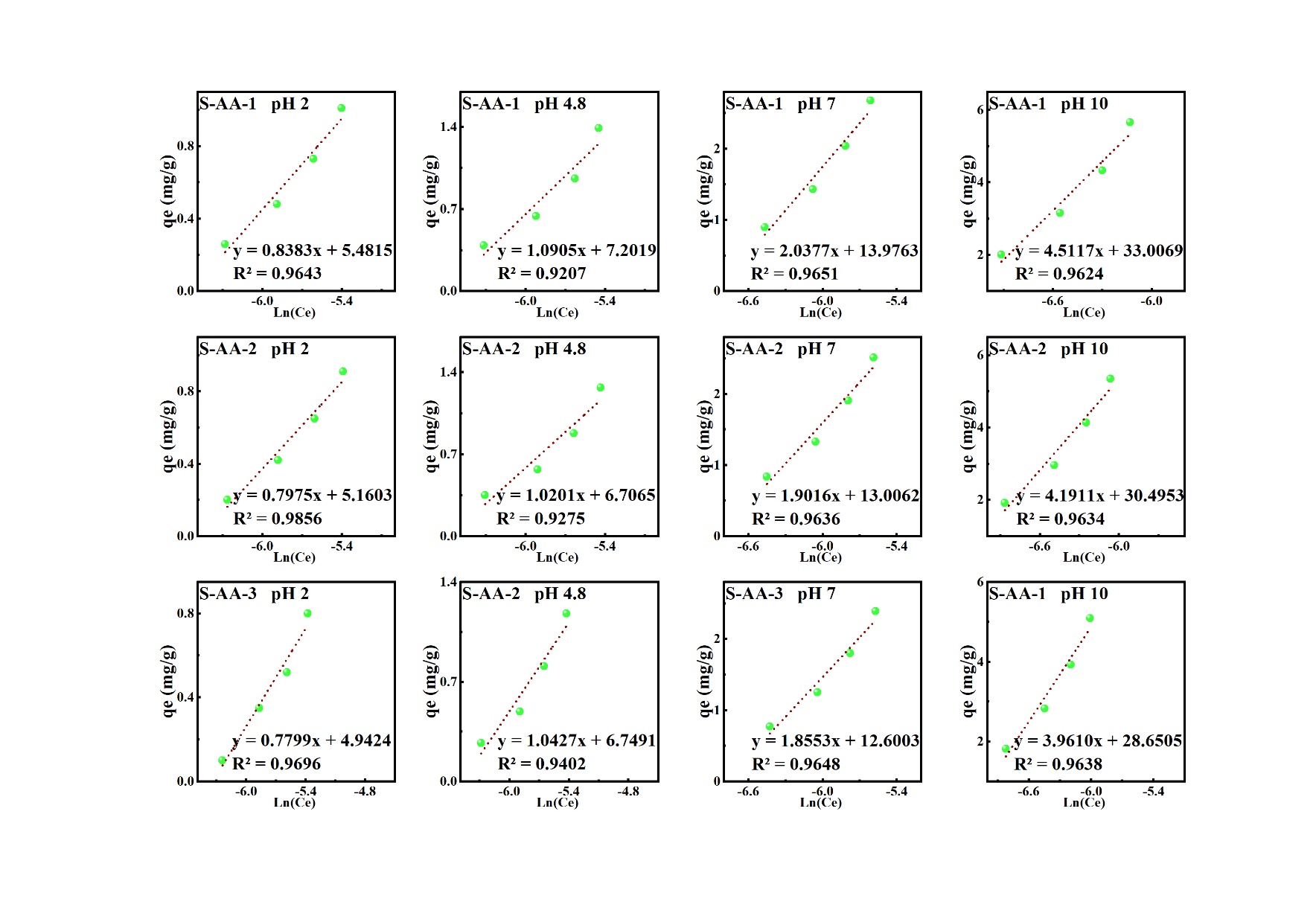


**Figure S17: Fitting the experimental data with the Temkin isotherm in different pH values**
